# An efficient heterogeneous catalyst (CuO@ARF) for on-water C-S coupling reaction: an application to the synthesis of phenothiazine structural scaffold

**DOI:** 10.1186/s13588-014-0017-7

**Published:** 2014-12-29

**Authors:** Debasish Sengupta, Basudeb Basu

**Affiliations:** Department of Chemistry, North Bengal University, Darjeeling, 734013 India

**Keywords:** C-S cross-coupling, CuO NPs, Heterogeneous catalyst, On-water reaction, Phenothiazine

## Abstract

**Background:**

Aryl sulfides have significant importance from biological and pharmaceutical aspects. Transition metal-catalyzed carbon-sulfur cross-coupling reaction represents an important tool for the synthesis of sulfides. Among various transition metals, copper salts or oxides have found vast applicability.

**Results:**

A simple procedure for the preparation of poly-ionic amberlite resins embedded with copper oxide nanoparticles (CuO NPs) (denoted as CuO@ARF) has been developed, characterized, and employed for the first time as a heterogeneous ligand-free catalyst for ‘on-water’ C-S cross-coupling reaction. The NPs of CuO with an average size (approximately 2.6 nm), as determined from high resolution transmission electron microscopy (HRTEM) images, are found to be a potentially active, chemoselective, and recyclable catalyst for the preparation of symmetrical and unsymmetrical aryl sulfides. Recycling of the catalyst was performed successfully for five consecutive runs, and apparently no leaching was observed in a hot filtration test. Excellent chemoselectivity between iodo- and bromo-arene has been exploited in step-wise C-S and C-N couplings to synthesize bioactive heterocyclic scaffold phenothiazine.

**Conclusions:**

An efficient method is established for the C-S cross-coupling reaction using heterogeneous catalyst CuO@ARF under ligand-free on-water condition. The catalyst is highly chemoselective among different aryl halides, which has been demonstrated in the synthesis heterocyclic scaffold phenothiazine. Furthermore, it is recyclable for five consecutive runs examined.

**Graphical abstract:**
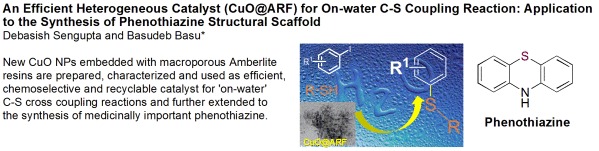
On-water C-S coupling using new heterogeneous nano-catalyst (CuO@ARF)

**Electronic supplementary material:**

The online version of this article (doi:10.1186/s13588-014-0017-7) contains supplementary material, which is available to authorized users.

## Background

**Figure 1:**
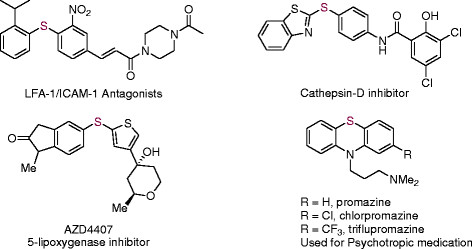
**Some biologically active compounds bearing C-S bond.**

The carbon-sulfur bonds are prevalent present in numerous medicinally important natural products [1]-[3]. Indeed, a number of drugs in various therapeutic uses such as HIV, cancer, diabetes, inflammatory, Alzheimer's, and Parkinson's diseases contain the aryl sulfide functional group [4]-[7]. A few biologically active compounds possessing a C-S bond are represented in Figure 1. For example, phenothiazines are an important class of organic compounds finding wide applications as drugs, insecticides, inhibitors of polymerization, antioxidants, paints, spectroscopic probes, etc. [8]. Therefore, in the recent era, the sp^2^C-S bond formation has been the subject of intense study in organic synthesis and medicinal chemistry, and researchers develop diverse mild cross-coupling methodologies for these pharmaceuticals.

The conventional methods for the C-S bond formation involve reduction of aryl sulfones or aryl sulfoxides using strong reducing agents like DIBAL-H or LiAlH_4_[9]. Besides, on-water C-S bond formation has been reported via thiol addition to α,β-unsaturated carbonyl compounds at room temperature [10]. In 1980, Migita et al. first showed the Pd-catalyzed thiation of aryl bromides using Pd(PPh_3_)_4_[11]. Subsequently, other metals like nickel [12],[13], copper [12],[14], cobalt [15], iron [16], rhodium [17], manganese [18], and indium [19] have also been employed, though in much less extent, as compared to other C-X (X = C, O, N, P) coupling reactions. This is possibly due to the notion that sulfur might act as the poison to suppress the catalytic activity through strong coordinating and adsorptive properties [20]. However, the last two decades have witnessed several new transition metal-based catalytic systems for the C-S coupling reactions between aryl halides and thiols. Amongst various transition metals, copper has been considered as the most useful for the C-S coupling reactions due to its special redox properties and cost-effectiveness. Many strategies have been successful by using homogeneous copper salts in the presence of suitable electron-rich and precious ligands for the C-S coupling reactions [21],[22].

On the other hand, nano-structured materials with high surface areas have been investigated as effective catalysts for various organic coupling reactions [23]. Catalysis of organic reactions by metal NPs supported on a suitable polymeric matrix offers the advantages of high catalytic activity, simplified isolation of the product, easy recovery, and recycling of the catalyst. Copper oxide nanoparticles (CuO NPs) are a good choice and indeed useful catalyst in the C-S coupling reaction between aryl halide and thiols [24]-[26]. However, previous on-water C-S coupling reactions involving copper species like CuI-TBAB [27], CuCl [28], or other metal species like Bi_2_O_3_[29], CoCl_2_^.^6H_2_O [30], or FeCl_3_^.^6H_2_O-bipyridyl complexes [31], etc. afford thioethers without easy recovery of the catalyst and recyclability. Direct use of CuO either in bulk or NPs requires organic solvents other than water and gave relatively poor yields in C-S coupling reaction. Considering our experience in the field of developing polymer-supported metal NPs as the heterogeneous catalyst in various coupling reactions, [32],[33] and in conjunction with our interest in the synthesis of various biologically important heterocyclic scaffolds mediated over solid supports, [34],[35] we were interested to develop polymer-supported CuO NPs and to use it as the catalyst in ‘on-water’ C-S coupling reaction between aryl halide and thiols.

We report herein our studies that constitute simple preparation and characterization of poly-ionic amberlite resins embedded with CuO NPs (CuO@ARF), which efficiently catalyze the C-S cross-coupling reaction under on-water and ligand-free conditions. Further application of this catalyst has been demonstrated in the synthesis of phenothiazine - an important structural motif of several potentially useful drugs and also used as chemosensors [36].

## Methods

Amberlite resin formate (ARF) was prepared from commercially available inexpensive amberlite resin chloride by an ion-exchange process as reported from this laboratory [37]. A mixture of ARF resin beads and Cu(OAc)_2_^.^H_2_O in DMF (50 mg g^−1^ of ARF) was heated at 110°C in a Teflon-capped sealed tube for 30 min with occasional gentle shaking. White ARF beads turned brownish in color during the process. Finally, these resin beads were filtered off, washed with water and acetone, dried, and characterized by Fourier transform infrared spectroscopy (FT-IR), X-ray diffraction (XRD), and transmission electron microscopy (TEM) analysis, and the copper content of the composite was estimated by atomic absorption spectroscopy (AAS). The XRD and TEM analyses suggested the presence of CuO NPs distributed on the surface of ARF, and we referred the as-synthesized nanocomposite material as CuO@ARF.

## Results and discussion

**Figure 2:**
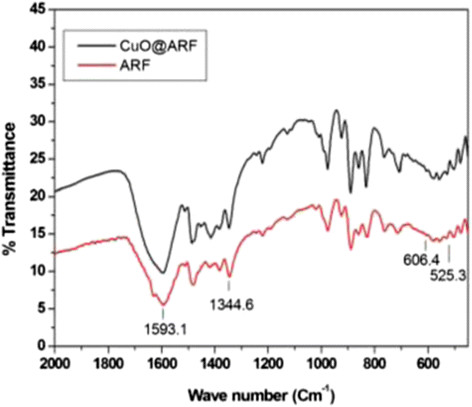
**FT-IR spectra of the ARF and CuO@ARF.**

At first, the presence of copper in the resin composite was measured by the AAS study. For this purpose, the sample (100 mg) was digested with concentrated HNO_3_ (1 mL) and Cu content was estimated to be 0.145 mmol g^−1^ of the resin-copper nanocomposite. The FT-IR spectra of CuO@ARF were recorded and compared with those of ARF (Figure 2). In the case of a similar heterogeneous Pd or bimetallic Pd/Cu nanocomposites, as developed from this laboratory, a significant increase in the stretching frequency of the carboxylate anion (HCOO¯) was observed [32],[33]. Here, apparently no change of symmetric and anti-symmetric stretching vibrations (for the HCOO¯) was observed and appeared respectively at about 1,345 and 1,593 cm^−1^ for both ARF and CuO@ARF species (Figure 2). However, the specific IR absorption bands of CuO at 606 and 525 cm^−1^ might overlap with those of ARF in the similar region [38].

**Figure 3:**
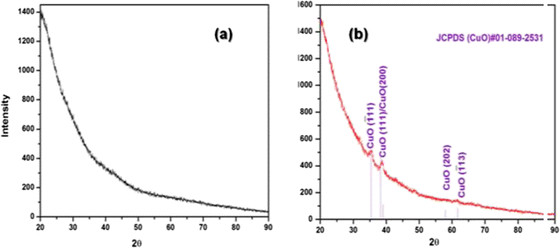
**XRD patterns of (a) ARF and (b) CuO embedded on the surface of amberlite resin formate (CuO@ARF).**

**Figure 4:**
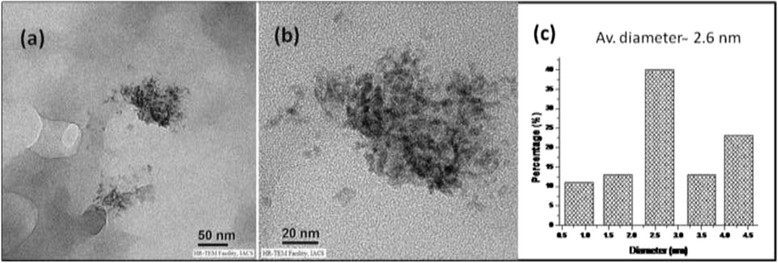
**TEM images of CuO@ARF. (a)** Scale bar 50 nm; **(b)** 20 nm; **(c)** average particle size from **(b)**.

We therefore examined the powder XRD patterns of ARF and those of CuO@ARF, as shown in Figure 3. The ARF resins are amorphous in nature, and the Bragg diffraction patterns of CuO@ARF indicate the presence of cupric oxide (CuO). The JCPDS peak positions of the CuO@ARF nanocomposite with relative intensities are in good agreement (Figure 3b). The presence of XRD peaks at 2*θ* of 35.39°, 38.29°, 38.99°, 57.91°, and 61.64° suggested the presence of CuO ($\bar{1}$ 11), (111), (200), (202), and ($\bar{1}$ 13) planes, respectively (JCPDS#01-089-2531). Further examination of high resolution TEM (HRTEM) images of the CuO@ARF at different magnifications (Figure 4a,b) suggested that the CuO NPs are embedded on the resin polymeric matrices with an average size around 2.6 nm (Figure 4c).

### Catalytic activity of CuO@ARF

In order to evaluate the catalytic activity of this newly developed nanocomposite (CuO@ARF) in C-S cross-coupling reaction between aryl halide and thiol, we performed a model study by taking a mixture of 4-iodoanisole and benzenethiol (in 1:1.2 ratios) and the catalyst (200 mg mmol^−1^ of aryl iodide) in water (Table 1). The first reaction in the presence of a base (K_2_CO_3_) at 100°C did afford the desired unsymmetrical sulfane but with a modest yield (62%) (Table 1, entry 1). Considering that the modest conversion might be due to the poor solubility of aryl iodide in water, we used an additive - tetrabutylammonium bromide (TBAB), in equimolar quantity. This afforded the corresponding thioether in fairly good yield (83%; entry 2). Further improvement was achieved by using the catalytic amount of sodium dodecyl sulfate (SDS; 10 mol%), [39] which furnished the desired product in 90% isolated yield (entry 3). While there was no cross-coupling observed at room temperature, reaction carried out at 60°C or in the absence of the base produced the sulfane in 56% to 68% isolated yields (entries 4 to 6). We also tried the reaction in DMF at 100°C, which did not produce the desired sulfane in very good yield (entry 7). In each case, a small quantity of the diphenylsulfide (≤5%) as the side product was also formed.

**Table 1:** **Optimization of reaction condition for the C-S cross-coupling using CuO@ARF** ^**a**^

Entry	Solvent	Base	Additive	Temperature/time	Yield (%)^b,c^
1	Water	K_2_CO_3_	-	100°C/24 h	62
2^d^	Water	K_2_CO_3_	TBAB	100°C/8 h	83
*3* ^*e*^	*Water*	*K*_*2*_ *CO*_*3*_	*SDS*	*100°C/8 h*	*90*
4^e^	Water	K_2_CO_3_	SDS	RT/24 h	00
5^e^	Water	K_2_CO_3_	SDS	60°C/24 h	68
6^e^	Water	-	SDS	100°C/24 h	56
7	DMF	K_2_CO_3_	-	100°C/24 h	67

^a^4-Iodoanisole (1 mmol), benzenethiol (1.2 mmol), CuO@ARF (200 mg), K_2_CO_3_ (1.1 mmol), and solvent (3 mL). ^b^Isolated yield. ^c^Small quantity of diphenyl disulfide was formed (≤5%). ^d^TBAB (1 eqv) was used. ^e^SDS (10 mol%) was used.

### Minimum loading of the catalyst

**Figure 5:**
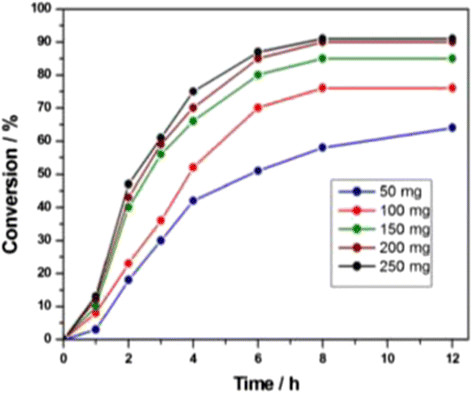
**Time conversion plots for the C-S cross-coupling between 4-iodoanisole and thiophenol.**

After optimizing the reaction conditions, we examined the minimum amount of the catalyst loading that is required to obtain effective conversion to sulfane. Experiments were performed with 4-iodoanisole (1 mmol) in the presence of varying quantities of CuO@ARF (from 50 mg to 250 mg; i.e., 0.46 mg to 2.30 mg of copper mmol^−1^ of iodoarene) and the results are shown in Figure 5. Conversions to the thioether at different time intervals were measured by high-performance liquid chromatography (HPLC) and the plot of percent conversion (±2%) versus time (h) revealed that minimum 200 mg of the catalyst (approximately 1.8 mg of copper) mmol^−1^ of iodoarene can give rise to the best result. In each case, reaction was continued up to 24 h and HPLC analysis showed no appreciable changes in the conversion (Figure 5 was plotted up to 12 h).

The optimized reaction conditions were then employed with different iodoarenes and other aromatic halides to couple with variety of aryl thiols to examine the scope and limitations of our catalyst for on-water C-S cross-coupling reaction. In general, it was observed that the cross-coupling reaction was successful producing corresponding diaryl sulfide in good to excellent yields. The results are presented in Table 2. Iodoarenes with both activated (methoxy, methyl) and deactivated functional groups (nitro or acetyl) effectively underwent C-S cross-coupling with different aryl thiols (entries 1 to 5). On the other hand, similar coupling reactions with bromo- and chlorobenzenes, including *p*-chloroacetophenone were not successful (entries 6 and 7). Increasing the catalyst loading and changing the base did not facilitate the C-S cross-coupling of *p*-bromotoluene (entry 8). The selectivity however might be useful for substrates bearing different halides. Thus, iodobromoarenes and iodochloroarene were subjected to coupling with aryl thiols in the presence of this heterogeneous catalyst affording highly chemoselective iodo-coupled product only (entries 9 to 11). Substituted thiophenol like 2,5-dimethylbenzenethiol underwent smooth reactions giving the corresponding unsymmetrical diarylsulfide in 89% isolated yield (entry 12). Aliphatic thiols also gave cross-coupling products without any difficulty but relatively in lower yields as compared to aromatic thiols (entries 13 to 15), which might be attributable to the fact that aliphatic thiols are less reactive than aromatic thiols [40]. Bis-couplings were performed equally efficiently starting from 1,3-diiodobenzene and *p*-tolylthiol and affording the desired 1,3-bis(*p*-tolylthio)benzene as the only product in 79% isolated yield (entry 16). It may be mentioned that only benzenethiol and 4-tolylthiol gave a minor quantity of corresponding disulfides during the reaction (≤5%), while in other cases, no disulfide was detected on thin-layer chromatography (TLC) or by HPLC analysis.

**Table 2:** **CuO@ARF-catalyzed C-S cross-coupling reactions between haloarenes and thiol** ^**a**^

Entry	Aryl halide	Thiol	Time (h)	Product	Yield (%)^b^
1	(4-H_3_CO)C_6_H_4_I	C_6_H_5_SH	8	(4-H_3_CO)H_4_C_6_ - S - C_6_H_5_	90
2	(3-H_3_CO)C_6_H_4_I	(4-C1)C_6_H_4_SH	12	(3-H_3_CO)H_4_C_6_ - S - C_6_H_4_(4-C1)	85
3	(2-H_3_CO)C_6_H_4_I	(4-CH_3_)C_6_H_4_SH	18	(2-H_3_CO)H_4_C_6_ - S - C_6_H_4_(4-CH_3_)	75
4	(3-NO_2_)C_6_H_4_I	(4-CH_3_)C_6_H_4_SH	11	(3-NO_2_)H_4_C_6_ - S - C_6_H_4_(4-CH_3_)	78
5	(4-H_3_CO)C_6_H_4_I	(4-CH_3_)C_6_H_4_SH	10	(4-H_3_CO)H_4_C_6_ - S - C_6_H_4_(4′-CH_3_)	75
6	(4-H_3_C)C_6_H_4_Br	C_6_H_5_SH	24	No reaction	-
7	(4-CH_3_CO)C_6_H_4_C1	C_6_H_5_SH	24	No reaction	-
8^c^	(4-H_3_C)C_6_H_4_Br	C_6_H_5_SH	24	No reaction	-
9	(5-Br)(2-H_3_CO)C_6_H_3_I	C_6_H_5_SH	8	(5-Br)(2-H_3_CO)H_3_C_6_ - S - C_6_H_5_	90
10	(3-Br)C_6_H_4_I	(4-CH_3_)C_6_H_4_SH	16	(3-Br)H_4_C_6_ - S - C_6_H_4_(4-CH_3_)	79
11	(3-C1)C_6_H_4_I	(4-CH_3_)C_6_H_4_SH	16	(3-C1)H_4_C_6_ - S - C_6_H_4_(4-CH_3_)	79
12	(4-H_3_CO)C_6_H_4_I	(2,5-(CH_3_)_2_)C_6_H_4_SH	7	(4-H_3_CO)H_4_C_6_ - S - C_6_H_3_(2,5-(CH_3_)_2_)	89
13	(4-H_3_CO)C_6_H_4_I	CySH	16	(4-H_3_CO)H_4_C_6_ - S - Cy	70
14	(4-H_3_CO)C_6_H_4_I	*n*-C_5_H_11_SH	16	(4-H_3_CO)H_4_C_6_ - S - C_5_H_11_-*n*	73
15	(3-H_3_CO)C_6_H_4_I	*n*-C_7_H_11_SH	16	(4-H_3_CO)H_4_C_6_ - S - C_7_H_15_-*n*	76
16^d^	1,3-C_6_H_4_I_2_	(4-CH_3_)C_6_H_4_SH	14	1,3-((4-CH_3_)C_6_H_4_S)_2_ - C_6_H_4_	79
17	(2-Br)C_6_H_4_I	(2-NH_2_)C_6_H_4_SH	8	(2-Br)H_4_C_6_ - S - C_6_H_4_(2-NH_2_)	82

^a^Aryl halide: thiol: catalyst (1 mmol: 1.2 mmol: 200 mg), SDS (10 mol%), and K_2_CO_3_ (1.1 mmol) were taken in water (3 mL) and heated at 100°C. ^b^Yield refers to pure isolated products characterized by spectroscopic (^1^H and ^13^C NMR) data. ^c^Catalyst (300 mg mmol^−1^ of aryl bromide), SDS (10 mol%), and Cs_2_CO_3_ (1.1 mmol). ^d^Aryl halide: thiol: catalyst (0.5 mmol: 1.2 mmol: 200 mg), SDS (10 mol%), and K_2_CO_3_ (1.1 mmol) were taken in water (3 mL).

As reported previously [26], the mechanism of the resin embedded CuO-catalyzed C-S coupling reaction is believed to proceed through the oxidative addition followed by reaction with thiol and then reductive elimination steps (Cu^II^ → Cu^III^ → Cu^II^). The role of the additive SDS is presumably to solubilize the organic substrates in an aqueous medium [41],[42]. Further beneficial assistance of the ‘microreactors’ formed by the surfactant like SDS in water medium organic reactions, as observed in other cases, cannot be ruled out [43]. In addition, the role of water might be attributed to the H-bonding (HB) effect, as reported previously on other occasions [44], has also been noticed in our cases. Thus, we isolated the cross-coupled product in higher yield performing the reaction in an aqueous medium as compared to in DMF (Table 1, entries 3 and 7).

**Figure 6:**
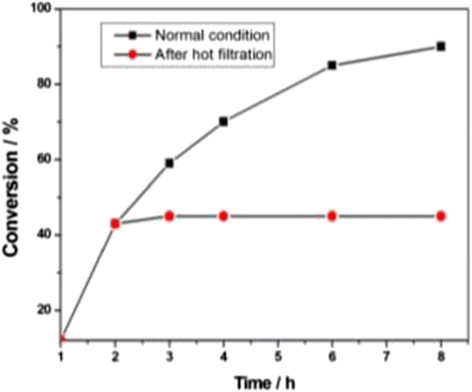
**Comparison of normal time profile with that of hot filtration test.** Conversions (±2%) at different time intervals for each plot were measured by HPLC.

Leaching of any metallic species from the amberlite resin polymeric matrices during or after the reaction was tested following the literature procedure [33],[45]. Accordingly, the reaction mixture of the C-S cross-coupling between 4-iodoanisole and thiophenol is filtered off to separate out the solid catalysts after 2 h in hot condition, and the filtrate was analyzed by HPLC (approximately 43% conversion). The AAS analysis of the filtrate solution did not show the presence of any copper species. Moreover, further continuing the reaction by heating the liquid phase at 100°C for another 6 h in the absence of any added catalyst (CuO@ARF) did not afford any appreciable conversion (HPLC analysis). These observations signify that any copper species might not be leached out from the heterogeneous support during the initial 2 h of the reaction (Figure 6).

**Figure 7:**
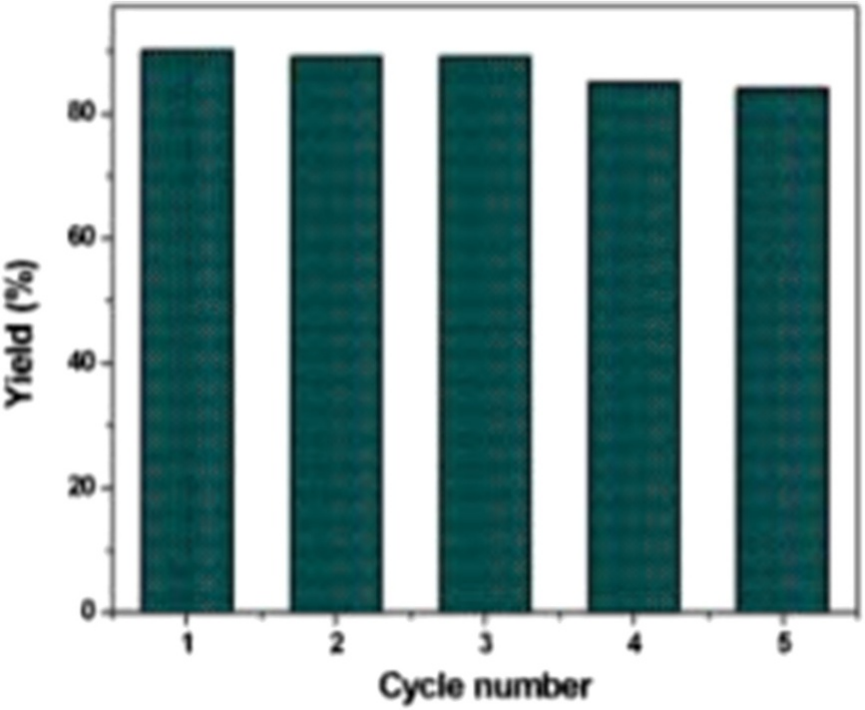
**Recycling experiments using CuO@ARF in the C-S cross-coupling reaction between 4-iodoanisole and thiophenol.**

One of the important factors for a heterogeneous catalyst lies in its efficiency for recycling. We investigated the reusability of CuO@ARF in the reaction between 4-iodoanisole and thiophenol again as the model case. After completion of the reaction, resin beads were filtered off from the reaction mixture, washed thoroughly with water followed by acetone (two times), and then dried under vacuum. The catalyst was reused in five consecutive runs including the first run. Gratifyingly, there was no considerable loss of the catalytic efficiency observed (Figure 7).

**Scheme 1:**
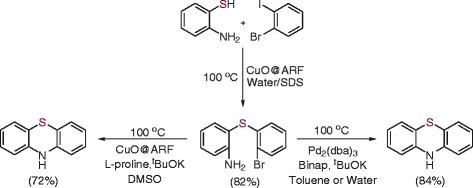
**Synthesis of phenothiazine.**

Since the present catalytic system is highly selective to aryl iodides only (i.e., the first oxidative addition to the sp^2^C-I bond), we decided to utilize this chemoselectivity between iodo- and bromo-substituents attached to the aryl moiety. Thus, a reaction of 1,2-bromoiodobenzene with 2-aminothiophenol in the presence of the catalyst CuO@ARF under similar conditions in water was performed. The C-S coupling occurred selectively at the sp^2^C-I bond, and we obtained 2-(2-bromophenylthio)benzene in 82% yield (Scheme 1). Further intramolecular Buchwald-Hartwig type coupling between amino-and bromo-substituents attached with two aromatic rings under Pd-BINAP catalyzed condition was carried out to afford phenothiazine in 84% isolated yield. The C-N coupling can also be accomplished by using the new catalyst (CuO@ARF) in the presence of L-proline as the ligand (Scheme 1) [46]. The ^1^H nuclear magnetic resonance (NMR) spectra run in DMSO-D6 and DMSO-D6-D_2_O confirmed formation of phenothiazine with the labile NH proton appeared at δ 8.5 ppm (see Additional file 1). We also performed the intramolecular Buchwald coupling in a water medium using both catalytic systems. Although the reaction works well with comparative yield of the phenothiazine in the case of using Pd_2_(dba)_3_, the use of CuO@ARF as the catalyst however does not give a satisfactory yield of the desired cyclic product.

A comparison chart of the efficiency of the present catalytic system with other metal-based ‘on-water’ catalytic systems reported in the literature showing advantages in terms of low loading, high efficiency, stability of the catalyst, recyclability, etc. has been presented in Table 3.

**Table 3:** **Comparison of various metal-based catalytic ‘on-water’ C-S coupling reactions with the present system CuO@ARF**

Entry	Catalytic system	Applicability	Remarks	Reference
01	CuI (1 mol%) 1.5 eqv TBAB (1 eqv); base KOH (1.5 eqv); 80°C, 10 h	With aryl iodide; bromo- and chloroarenes gave poor yields even using 5 mol% CuI	Excess strong base, not recyclable; CuI is poorly soluble in water, TBAB is moisture sensitive.	[27]
02	CuCl and 1,2-diamine as ligand (>2 eqv); 120°C overnight heating	With iodo- and bromoarenes. No selectivity was examined	Precious 1,2-diamines in >2, equivalents, long reaction time, recyclable using the recovered solution - catalyst was not separated.	[28]
03	Bi_2_O_3_/Diamine ligand; (each 10 mol%); 1 eqv KOH at 100°C	With aryl iodide	Presence of a ligand, high loading of the metal catalyst, long reaction time; recyclable using the recovered solution - catalyst was not separated.	[29]
04	CoCl_2_^.^6H_2_O/cationic 2,2′-bipyridyl system, 1 eqv KOH; excess zinc, 100°C	With aryl halides (iodide, bromide, and chloride).	Presence of cationic 2,2′-bipyridyl; excess Zn; long reaction time; recyclable using the recovered solution - catalyst was not separated.	[30]
05	FeCl_3_^.^6H_2_O (10 mol%) - bipyridyl complexes (10 mol%) ; KOH (4 eqv); 100°C, 24 h	With aryl iodide	Excess base, long reaction time; recyclable using the recovered solution - catalyst was not separated.	[31]
06	CuO@ARF; copper oxide (2.8 mol%); base K_2_CO_3_ (1.1 eqv); 100°C, 8 h	With aryl iodide	Mild base - nearly equivalent (1: 1.1); shorter reaction time; chemoselectivity between iodo and bromo has been utilized in the synthesis of medicinally important phenothiazine scaffold, easy separation of heterogeneous catalyst (by simple filtration of resin beads), recyclable for five runs without loss of activity; catalytic amount of SDS, ligand-free.	

## Conclusions

Our studies established that poly-ionic resin-supported CuO NPs (CuO@ARF) are an efficient catalyst in the C-S coupling reaction under ligand-free ‘on-water’ conditions. Low loading of the catalyst, recyclability without leaching, and chemoselectivity amongst aromatic halides are notable features. Further application of the chemoselectivity has been demonstrated in the synthesis of bioactive heterocyclic scaffold phenothiazine. Considering the inexpensive catalytic system along with the application to the synthesis of medicinally important structural scaffold, this heterogeneous catalyst and greener method can find wider applications in organic synthesis.

### Experimental

Amberlite IRA 900 (chloride form) was purchased from Acros Organics, Geel, Belgium, and used after washing with water and acetone followed by drying under vacuum. Cupric acetate was purchased from S.D. Fine Chem. Limited, Mumbai, India, and other chemicals were purchased and used directly. For column chromatography, silica (60 to 200 μm) (Sisco Research Laboratories, Mumbai, India), and for TLC, Merck plates coated with silica gel 60, F254 were used (Merck & Co., Inc., Whitehouse Station, USA). FT-IR spectra were recorded in FT-IR-8300 Shimadzu spectrophotometer (Shimadzu, Kyoto, Japan). NMR spectra were taken in CDCl_3_ using Bruker Avance AV-300 spectrometer (Bruker AXS, Inc., Yokohama-shi, Japan) operating for ^1^H at 300 MHz and for ^13^C at 75 MHz. The spectral data were measured using TMS as the internal standard (for ^1^H) and CDCl_3_ (for ^13^C). AAS measurements were made using Varian SpectrAA 50B instrument (Varian Medical Systems, Melbourne, Australia). Progress of the reaction was monitored by HPLC (1260 Infinity, Agilent Technologies, Santa Clara, USA), column: ZORBAX Rx-SIL (4.6 × 150 mm, 5 μm), eluent: n-hexane (flow rate 2 mL min^−1^). The XRD studies of the powder samples were done using the Rigaku SmartLab (Shibuya-ku, Japan) (9 kW) diffractometer using CuKα radiation. HRTEM of the samples was recorded with JEOL JEM-2100 F (FEG) (JEOL Ltd., Akishima-shi, Japan) operating at 200 kV.

### Preparation of CuO@ARF

To a solution of Cu(OAc)_2_,^.^H_2_O (50 mg, 0.25 mmol) in DMF (5 mL) was added ARF (1 g), and the mixture taken in a Teflon-capped sealed tube was heated at 110°C for 30 min with occasional gentle shaking. The supernatant liquid became completely colorless by this time, and the greyish beads of ARF visibly turned brownish. The mixture was cooled to room temperature, and the resin beads were filtered off and washed repeatedly with water (3 × 5 mL) and acetone (2 × 5 mL). Resulting brown beads were dried under vacuum and used for analyses and evaluation of catalytic activity.

### General conditions for C-S cross-coupling reaction

To a suspension of CuO@ARF (200 mg) in water (3 mL) were added aryl halide (1 mmol), thiol (1.2 mmol), K_2_CO_3_ (1.1 mmol), and SDS (10 mol%), and the reaction mixture was heated in a screw-capped sealed tube at 100°C for several hours as mention in Table 2. Proceeding of the reaction was monitored by TLC at time intervals. After completion, the mixture was cooled, diluted with water (5 mL), and then filtered through a cotton bed to separate out the resin beads. The resin beads were washed with ethyl acetate (2 mL), and the filtrate was extracted with ethyl acetate (3 × 10 mL). The combined organic layer was dried over anhydrous Na_2_SO_4_ and concentrated under vacuum. The residue obtained was purified by column chromatography using light petroleum as the eluent. All products were identified on the basis of spectral data (^1^H and ^13^C NMR) and also compared with reported melting point (for solid compounds and as available) (see Additional file 1).

The resin beads separated out from the reaction mixture were successively washed with water (3 × 5 mL) and acetone (2 × 5 mL) and then dried under vacuum for use in the next batch of recycle run.

### Preparation of phenothiazine from selective iodo-coupled product (Table 2, entry 17) using Pd-BINAP catalyst

To a mixture of 2-(2-bromophenylthio)benzeneamine (1 mmol, 280 mg) in toluene were added ^t^BuOK (1.5 mmol, 168 mg), Pd_2_(dba)_3_ (2 mol%), and BINAP (4 mol%). The reaction mixture taken in a screw-capped sealed tube was heated at 100°C for 4 h. After cooling, the reaction mixture was diluted with water (5 mL) and extracted with ethyl acetate (3 × 10 mL). The combined extracts were washed with brine, dried (Na_2_SO_4_), and evaporated. The residue was purified over a silica gel column to obtain white crystals of phenothiazine (167 mg, 84%), characterized by ^1^H and ^13^C NMR, and compared with literature data (see Additional file 1).

10*H*-phenothiazine [46], m.p. 185°C to 186°C (Lit. m.p. 186°C to 187°C). ^1^H NMR (DMSO-d6, 300 MHz): *δ*/ppm 6.72 to 6.77 (*m*, 4H, ArH), 6.89 to 7.01 (*m*, 4H, ArH), 8.58 (*s*, 1H, NH) ^13^C NMR (DMSO-d6, 75 MHz): *δ*/ppm 114.4, 116.4, 121.8, 126.3, 127.5, and 142.1.

### Preparation of phenothiazine from selective iodo-coupled product (Table 2, entry 17) using CuO@ARF catalyst

To a mixture of 2-(2-bromophenylthio)benzeneamine (1 mmol, 280 mg) in dimethyl sulfoxide were added ^t^BuOK (1.5 mmol, 168 mg), CuO@ARF (200 mg, 1.8 mg of copper, 2.83 mol% Cu) and L-proline (5.66 mol%). The reaction mixture taken in a screw-capped sealed tube was heated at 100°C for 48 h. After cooling, the reaction mixture was diluted with water (5 mL) and extracted with ethyl acetate (3 × 10 mL). The combined extracts were washed with brine, dried (Na_2_SO_4_), and evaporated. The residue was purified over a silica gel column to obtain white crystals of phenothiazine (143 mg, 72%), characterized by ^1^H and ^13^C NMR and compared with literature data (see Additional file 1).

## Additional file

## Electronic supplementary material

Additional file 1: **Supporting data.** ^1^H and ^13^C NMR spectral data for sulfanes and scanned spectra and a comparative ^1^H-NMR spectra of Phenothiazine. (PDF 552 KB)

Below are the links to the authors’ original submitted files for images.

Authors’ original file for figure 1

Authors’ original file for figure 2

Authors’ original file for figure 3

Authors’ original file for figure 4

Authors’ original file for figure 5

Authors’ original file for figure 6

Authors’ original file for figure 7

Authors’ original file for figure 8
