# Supplementary material for: An efficient heterogeneous catalyst (CuO@ARF) for on-water C-S coupling reaction: an application to the synthesis of phenothiazine structural scaffold
Source: Org Med Chem Lett. 2014 Dec 29;4:17. doi: 10.1186/s13588-014-0017-7 (PMC4970439; doi:10.1186/s13588-014-0017-7)
Supplement: Supplementary file 1 — Additional file 1: Supporting data. 1H and 13C NMR spectral data for sulfanes and scanned spectra and a comparative 1H-NMR spectra of Phenothiazine. (PDF 552 KB) [file 13588_2014_17_MOESM1_ESM.pdf]

Supporting data

An efficient heterogeneous catalyst (CuO@ARF) for on-water C-S coupling reaction: an application to the synthesis of phenothiazine structural scaffold

Debasish Sengupta and Basudeb Basu*

Department of Chemistry, North Bengal University, Darjeeling-734013, India

Fax: +91 353 2699001

E-mail: basu_nbu@hotmail.com

Table of Contents

S1. ^{1}H and ^{13}C NMR spectral data for sulfanes listed in Table 2.

S2. References.

S3. Scanned ^{1}H and ^{13}C NMR spectra of C-S cross-coupling products listed in Table 2 and Phnothiazine.

S4. A comparative ^{1}H-NMR spectra for Phenothiazine run in DMSO-d6 and DMSO-d6+D_2O.

S1. ^{1}H and ^{13}C NMR spectral data for sulfanes listed in Table 2.

Table 2, entry 1, (4–Methoxyphenyl)(phenyl)sulfane [1], colourless liquid. ^{1}H NMR ($CDCl_3$, 300 MHz): δ/ppm 3.81 (s, 3H, OCH_3), 6.89 (dd, *J* = 2.1 and 6.9 Hz, 2H, ArH), 7.13–7.25 (m, 5H, ArH), 7.41 (dd, *J* = 2.1, 6.6 Hz, 2H, ArH); ^{13}C NMR ($CDCl_3$, 75 MHz): δ/ppm 55.3, 114.9, 124.1, 125.7, 128.1, 128.9, 135.3, 138.6, 159.7.

Table 2, entry 2, (4–Chlorophenyl)(3–methoxylphenyl)sulfane [2], liquid. ^{1}H NMR ($CDCl_3$, 300 MHz): δ/ppm 3.75 (s, 3H, OCH_3), 6.80–6.81(m, 1H, ArH), 6.85–6.91(m, 2H, ArH), 7.20 (d, *J* = 8.1 Hz, 1H, ArH),7.24–7.26 (m, 4H, ArH); ^{13}C NMR ($CDCl_3$, 75 MHz): δ/ppm 55.2, 113.1, 116.2, 123.2, 129.3, 130.1, 132.3, 133.1, 134.1, 136.4, 160.1.

Table 2, entry 3, (2–Methoxyphenyl)(*p*–tolyl)sulfane [1], liquid. ^{1}H NMR ($CDCl_3$, 300 MHz): δ/ppm 2.35 (s, 3H, CH_3), 3.88 (s, 3H, OCH_3), 6.80–6.88 (m, 2H, ArH), 6.94 (dd, *J* = 1.5 and 7.8 Hz, 1H ArH), 7.13–7.20 (m, 3H, ArH), 7.31 (d, *J* = 8.1 Hz, 2H, ArH); ^{13}C NMR ($CDCl_3$, 75 MHz): δ/ppm 21.1, 55.8, 110.6, 121.2, 125.7, 127.4, 129.7, 129.8, 130.1, 132.9, 137.7, 156.5.

Table 2, entry 4, (3–Nitrophenyl)(*p*–tolyl)sulfane [3], pale yellow solid, m.p. 57–58 (Lit. m.p. 60–61 °C). ^{1}H NMR ($CDCl_3$, 300 MHz): δ/ppm 2.39 (s, 3H, CH_3), 7.22 (d, *J* = 9.0 Hz, 2H, ArH), 7.37–7.45 (m, 4H, ArH), 7.94–7.96 (m, 2H, ArH); ^{13}C NMR ($CDCl_3$, 75 MHz): δ/ppm 21.2, 120.4, 122.2, 127.8, 129.5, 130.7, 133.3, 134.1, 139.5, 141.6, 148.6.

Table 2, entry 5, (4–Methoxyphenyl)(*p*–tolyl)sulfane [4], liquid, ^{1}H NMR ($CDCl_3$, 300 MHz): δ/ppm 2.29 (s, 3H, CH_3), 3.79 (s, 3H, OCH_3), 6.86 (d, *J* = 9.0 Hz, 2H, ArH), 7.05 (d, *J* = 8.1 Hz, 2H, ArH), 7.13 (d, *J* = 8.1 Hz, 2H, ArH), 7.35 (d, *J* = 9.0 Hz, 2H, ArH); ^{13}C NMR ($CDCl_3$, 75 MHz): δ/ppm 20.9, 55.3, 114.8, 125.6, 129.3, 129.7, 134.3, 136.1, 159.4.

Table 2, entry 9, (5–Bromo–2–methoxyphenyl)(phenyl)sulfane [1], liquid. ^{1}H NMR ($CDCl_3$, 300 MHz): δ/ppm 3.84 (s, 3H, OCH_3), 6.73 (d, *J* = 8.4 Hz, 1H, ArH), 7.03 (d, *J* = 2.4 Hz, 1H,

ArH), 7.26 (dd, J = 2.4 and 8.7 Hz, 1H, ArH), 7.31–7.40 (m, 5H, ArH); ^{13}C NMR ($CDCl_3$, 75 MHz): δ/ppm 56.1, 112.1, 113.2, 127.7, 128.0, 129.4, 130.1, 132.1, 132.5, 132.7, 155.7.

Table 2, entry 10, (3–Bromophenyl)(*p*–tolyl)sulfane [5], liquid. ^{1}H NMR ($CDCl_3$, 300 MHz): δ/ppm 2.36 (s, 3H, CH_3), 7.10–7.18 (m, 4H, ArH), 7.24–7.28 (m, 1H, ArH), 7.32–7.35 (m, 3H, ArH); ^{13}C NMR ($CDCl_3$, 75 MHz): δ/ppm 21.2, 122.9, 127.2, 129.0, 129.4, 130.2, 130.3, 131.1, 133.2, 138.5, 140.3.

Table 2, entry 11, (3–Chlorophenyl)(*p*–tolyl)sulfane, liquid. ^{1}H NMR ($CDCl_3$, 300 MHz): δ/ppm 2.36 (s, 3H, CH_3), 7.07–7.19 (m, 6H, ArH), 7.34 (dd, J = 1.8 and 6.3 Hz, 2H, ArH); ^{13}C NMR ($CDCl_3$, 75 MHz): δ/ppm 21.2, 126.1, 126.7, 128.3, 129.4, 129.9, 130.3, 133.3, 134.8, 138.6, 140.0.

Table 2, entry 12, (4–Methoxyphenyl)(2,5–dimethylphenyl)sulfane [6], liquid. ^{1}H NMR ($CDCl_3$, 300 MHz): δ/ppm 2.20 (s, 3H, CH_3), 2.32 (s, 3H, CH_3), 3.80 (s, 3H, OCH_3), 6.85–6.92 (m, 4H, ArH), 7.06 (d, J = 7.5 Hz, 1H, ArH), 7.29 (dd, J = 2.1 and 6.6 Hz, 2H, ArH); ^{13}C NMR ($CDCl_3$, 75 MHz): δ/ppm 19.8, 20.9, 55.3, 114.9, 124.9, 127.2, 130.1, 130.2, 133.9, 134.3, 136.0, 136.1, 159.2.

Table 2, entry 13, Cyclohexyl(4–methoxyphenyl)sulfane [7], liquid. ^{1}H NMR ($CDCl_3$, 300 MHz): δ/ppm 1.21–1.94 (m, 10H, CH_2), 2.89 (m, 1H, S–CH), 3.80 (s, 3H, OCH_3), 6.83 (dd, J = 2.1 and 6.6 Hz, 2H, ArH), 7.36–7.40 (m, 2H, ArH); ^{13}C NMR ($CDCl_3$, 75 MHz): δ/ppm 25.8, 26.1, 33.4, 47.9, 55.3, 114.3, 125.1, 135.5, 159.3.

Table 2, entry 14, (4–Methoxyphenyl)(pentyl)sulfane [8], liquid. ^{1}H NMR ($CDCl_3$, 300 MHz): δ/ppm 0.88 (t, J = 7.2 Hz, 3H), 1.26–1.40 (m, 4H, CH_2–CH_2), 1.56–1.61 (m, 2H, CH_2), 2.81 (m, 2H, S–CH_2), 3.79 (s, 3H, OCH_3), 6.83 (dd, J = 2.1 and 6.6 Hz, 2H, ArH), 7.33 (dd, J = 2.1 and 6.6 Hz, 2H, ArH); ^{13}C NMR ($CDCl_3$, 75 MHz): δ/ppm 13.9, 22.2, 29.0, 30.8, 35.7, 55.3, 114.4, 126.9, 132.9, 158.6.

Table 2, entry 15, Heptyl(3–methoxyphenyl)sulfane, liquid. ^{1}H NMR ($CDCl_3$, 300 MHz): δ/ppm 0.85–0.90 (m, 3H, CH_3 and CH_2), 1.26–1.68 (m, 10H, CH_2), 2.91 (t, J = 7.2 Hz, 2H, S–CH_2), 3.79 (s, 3H, OCH_3), 6.696 (ddd, J = 0.9, 2.4 and 8.1 Hz, 1H, ArH), 6.85–6.91(m, 2H, ArH), 7.16–7.25 (m, 1H, ArH); ^{13}C NMR ($CDCl_3$, 75 MHz): δ/ppm 14.0, 22.6, 28.8, 29.1, 31.3, 31.7, 33.3, 55.2, 111.2, 114.0, 120.8, 129.6, 138.5, 159.8.

Table 2, entry 16, 1,3–Bis(*p*–tolylthio)benzene [1], white solid, m.p. 84–85 °C. ^{1}H NMR ($CDCl_3$, 300 MHz): δ/ppm 2.34 (s, 6H, CH_3), 6.98–7.01 (m, 2H, ArH), 7.06–7.13 (m, 6H, ArH), 7.27 (dd, J = 1.5 and 6.3 Hz, 4H, ArH); ^{13}C NMR ($CDCl_3$, 75 MHz): δ/ppm 21.1, 126.6, 129.0, 129.3, 130.1, 132.9, 138.0, 138.7.

Table 2, entry 17, 2–(2–Bromophenylthio)benzenamine [9], m.p. 63–64 °C (Lit. m.p. 62–63 °C). ^{1}H NMR ($CDCl_3$, 300 MHz): δ/ppm 4.13 (s, 2H, NH_2), 6.60 (dd, J = 1.5 and 7.8 Hz, 1H, ArH), 6.75–6.83 (m, 2H, ArH), 6.92–6.98 (m, 1H, ArH), 7.05–7.10 (m, 1H, ArH), 7.23–7.30 (m, 1H, ArH), 7.43–7.52 (m, 2H, ArH); ^{13}C NMR ($CDCl_3$, 75 MHz): δ/ppm 113.0, 115.5, 119.0, 120.6, 126.1, 126.2, 127.7, 131.7, 132.7, 137.79, 137.8, 148.9.

S2. References

[1] Basu B, Mandal B, Das S, Kundu S (2009) Catechol violet as new, efficient, and versatile ligand for Cu(I)-catalyzed C–S coupling reactions. Tetrahedron Lett 50:5523-5528.

[2] Kwong FY, Buchwald SL (2002) A general, efficient, and inexpensive catalyst system for the coupling of aryl iodides and thiols. Org Lett 4:3517-3520.

[3] Wu WY, Wang JC, Tsai FY (2009) A reusable $FeCl_3 \cdot 6H_2O$/cationic 2,2'-bipyridyl catalytic system for the coupling of aryl iodides with thiols in water under aerobic conditions. Green Chem 11:326-329.

[4] Jammi S, Sakthivel S, Rout L, Mukherjee T, Mandal S, Mitra R, Saha Punniyamurthy T (2009) CuO nanoparticles catalyzed C–N, C–O, and C–S cross-coupling reactions: scope and mechanism. J Org Chem 74:1971-1976.

[5] Ampbell JR (1962) Reduction of Disulfides with Copper. Preparation of some thioethers. J Org Chem 27: 2207-2209.

[6] Fernandez–Rodriguez MA, Shen Q, Hartwig JF (2006) Highly efficient a functional-group-tolerant catalysts for the palladium- catalyzed coupling of aryl chlorides with thiols. Chem Eur J 12:7782-7796.

[7] Kao HL, Chen CK, Wang YJ, Lee C-F (2011) An efficient copper-catalyzed cross-coupling reaction of thiols with aryl iodides. Eur J Org Chem 1776-1781.

[8] Suter CM, Hansen HL (1932) The preparation and germicidal properties of para-hydroxyphenyl alkyl sulphides. J Am Chem Soc 54:4100-4104.

[9] Ma D, Geng Q, Zhang H, Jiang Y (2010) Assembly of substituted phenothiazines by a sequentially controlled CuI/l-proline-catalyzed cascade C–S and C–N bond formation. Angew Chem Int Ed 49:1291-1294.

S3. Scanned ^{1}H and ^{13}C NMR spectra of C-S cross-coupling products listed in Table 2 and Phnothiazine.

Table 2, entry 1

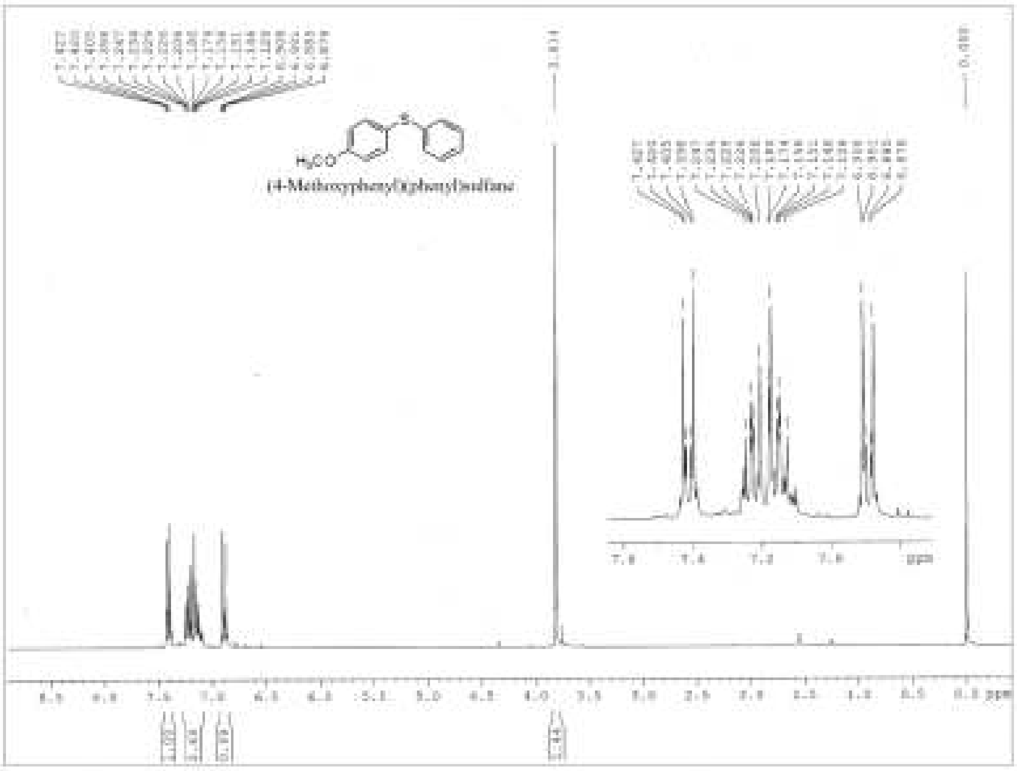

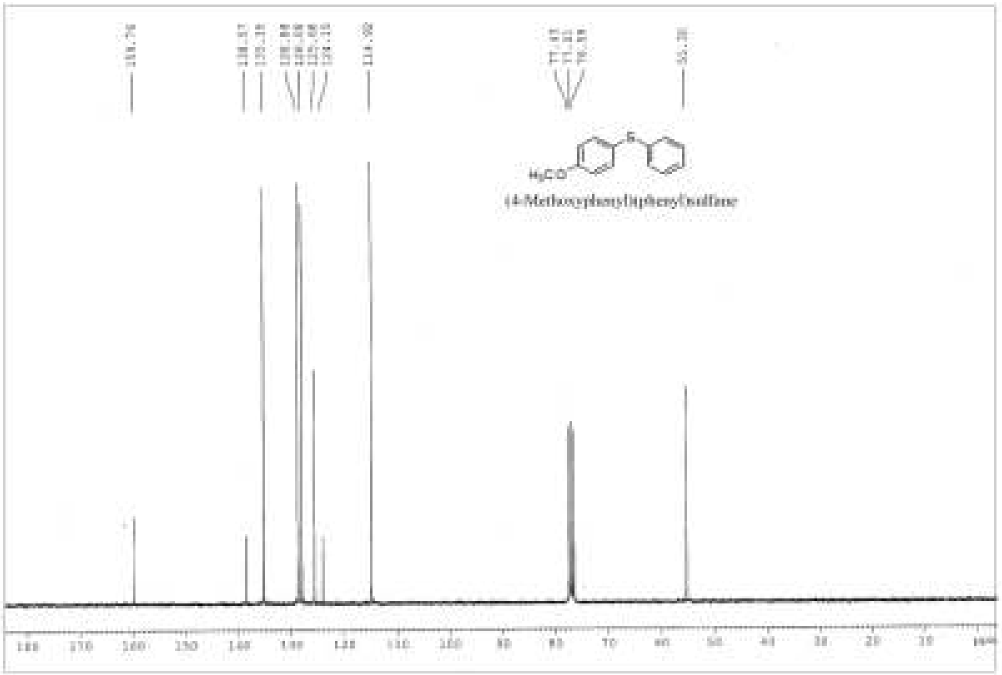

Table 2, entry 2

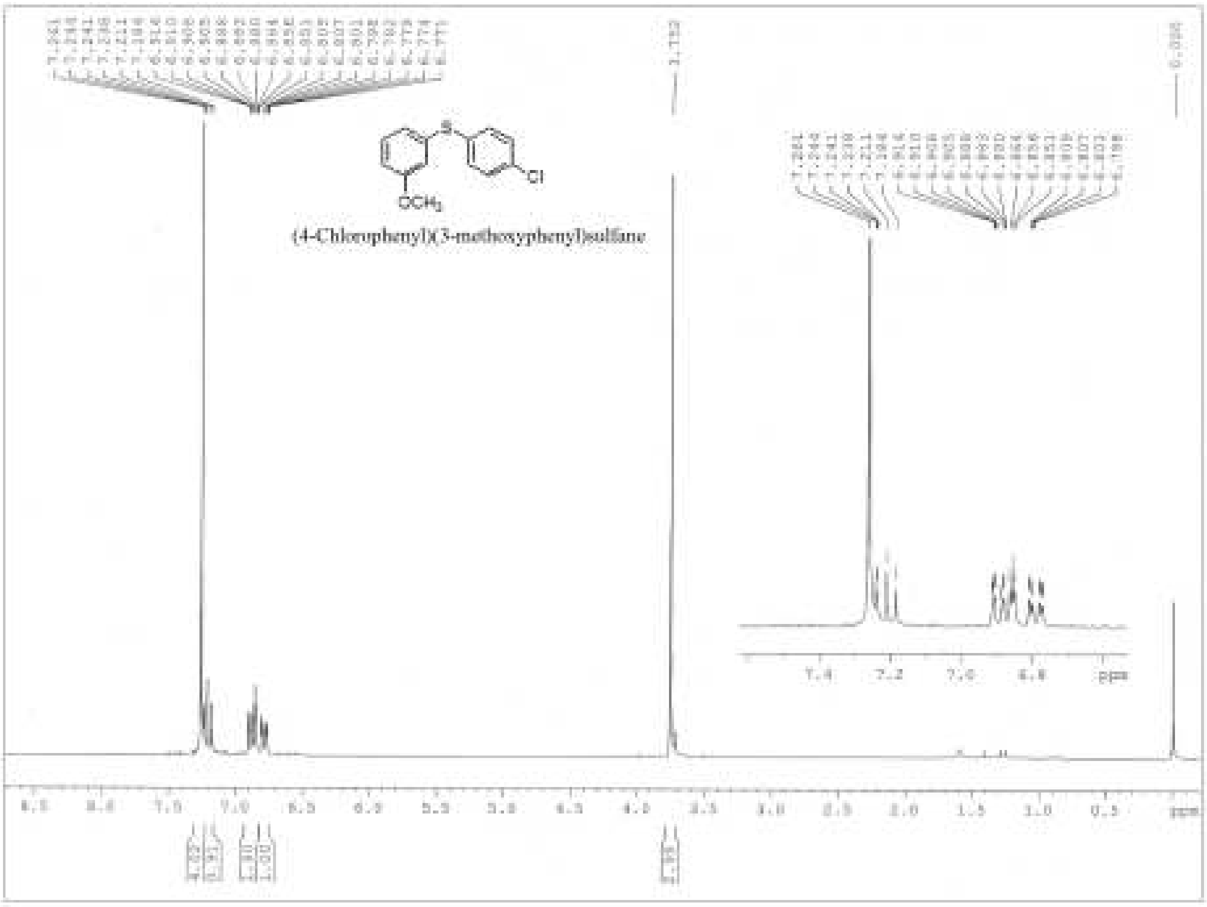

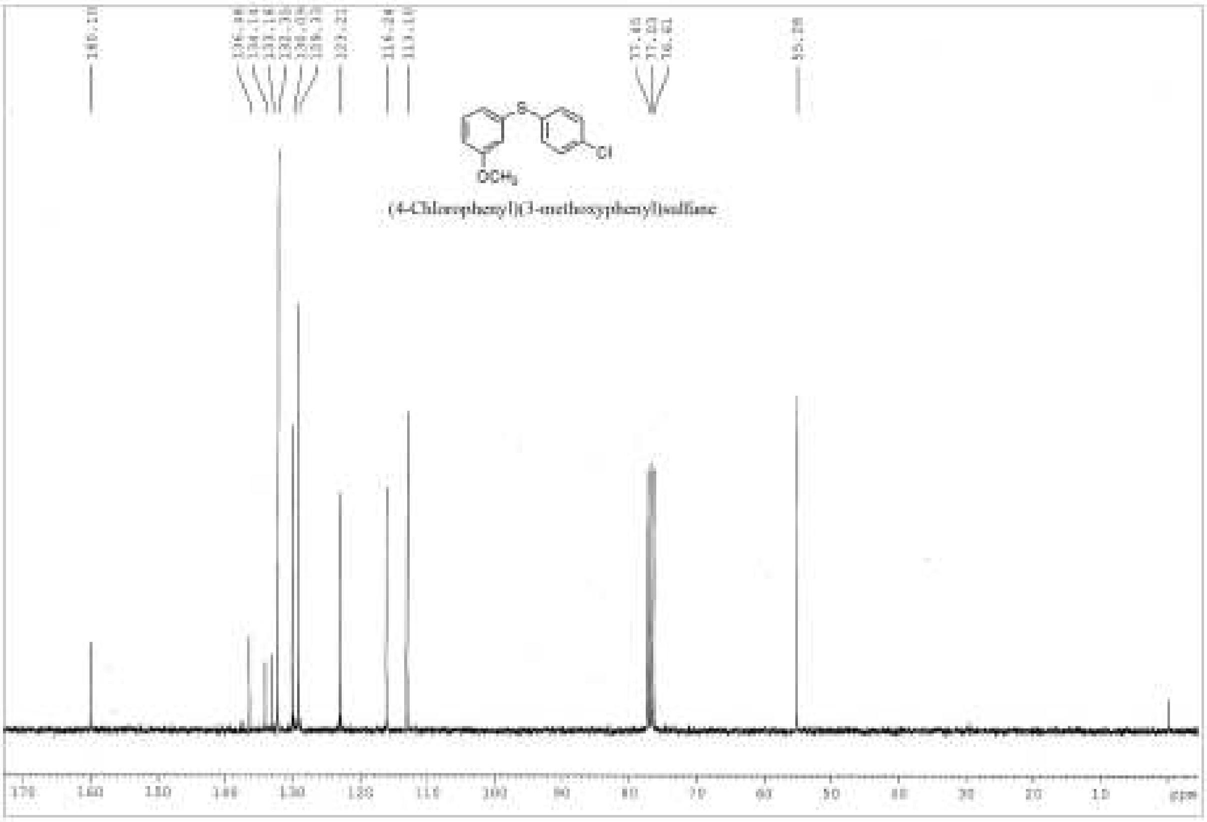

Table 2, entry 3

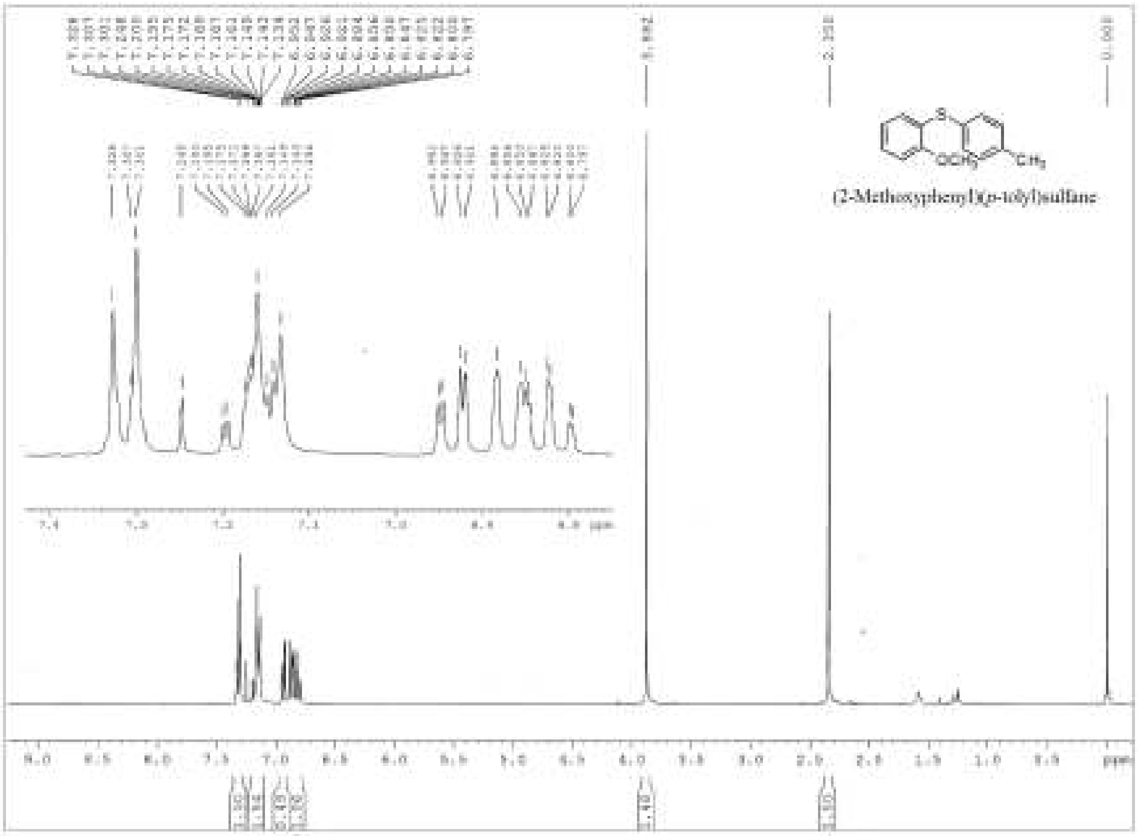

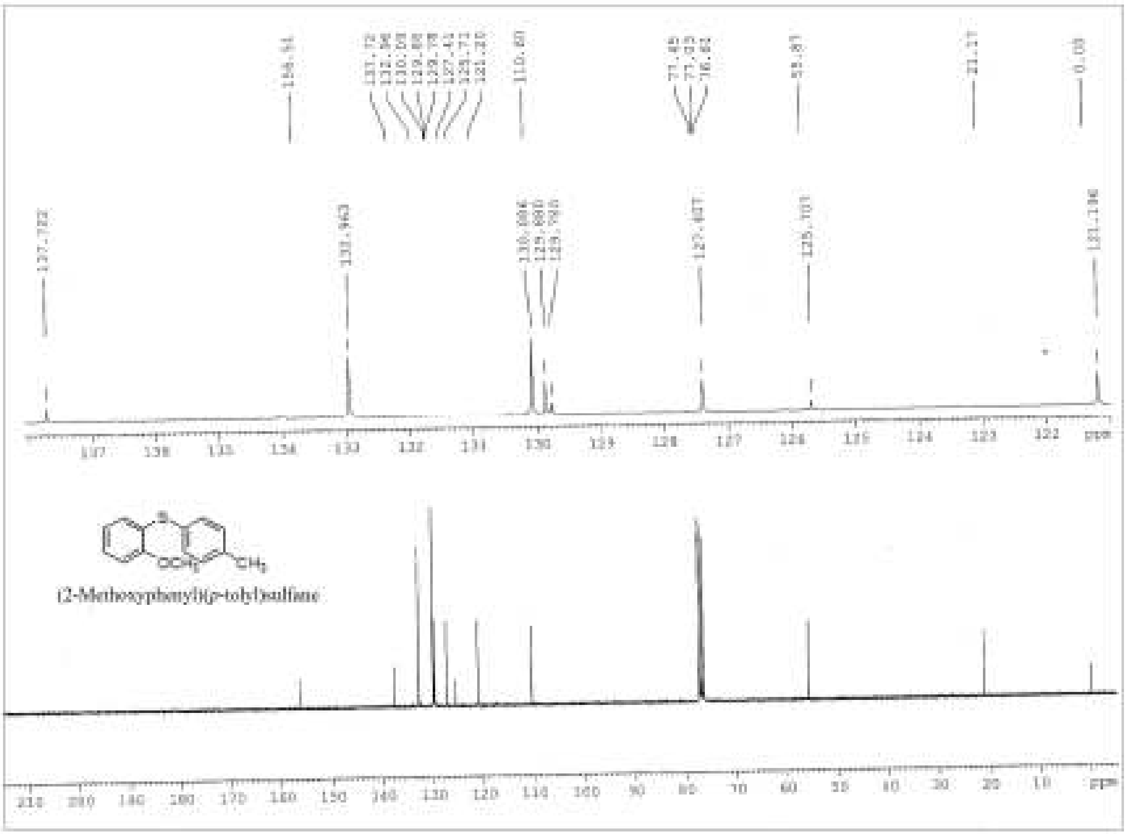

Table 2, entry 4

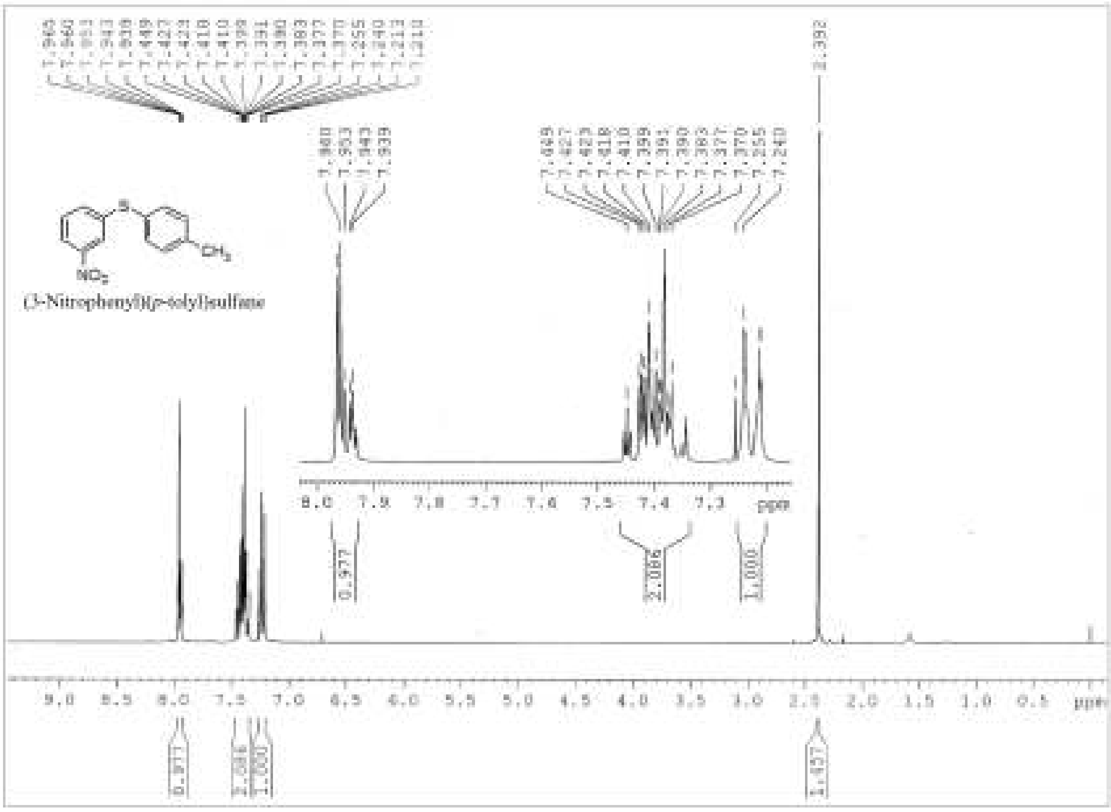

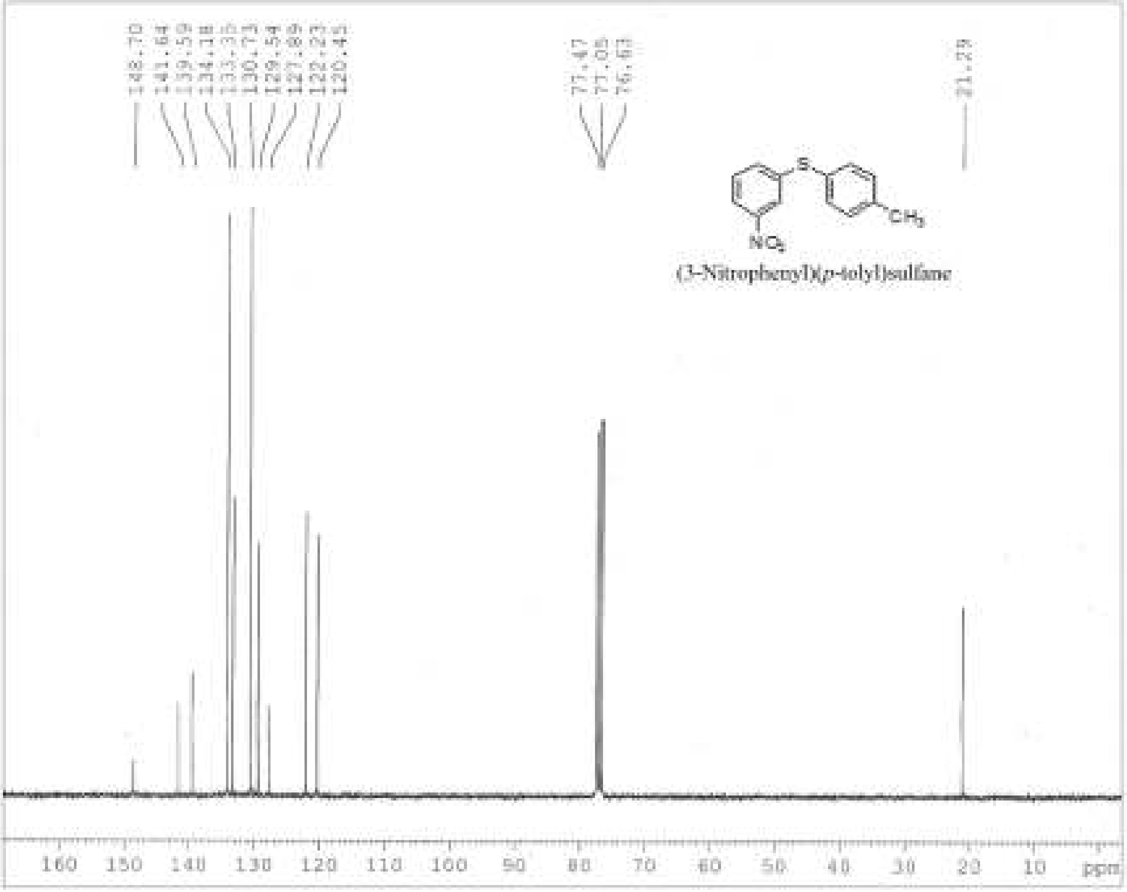

Table 2, entry 5

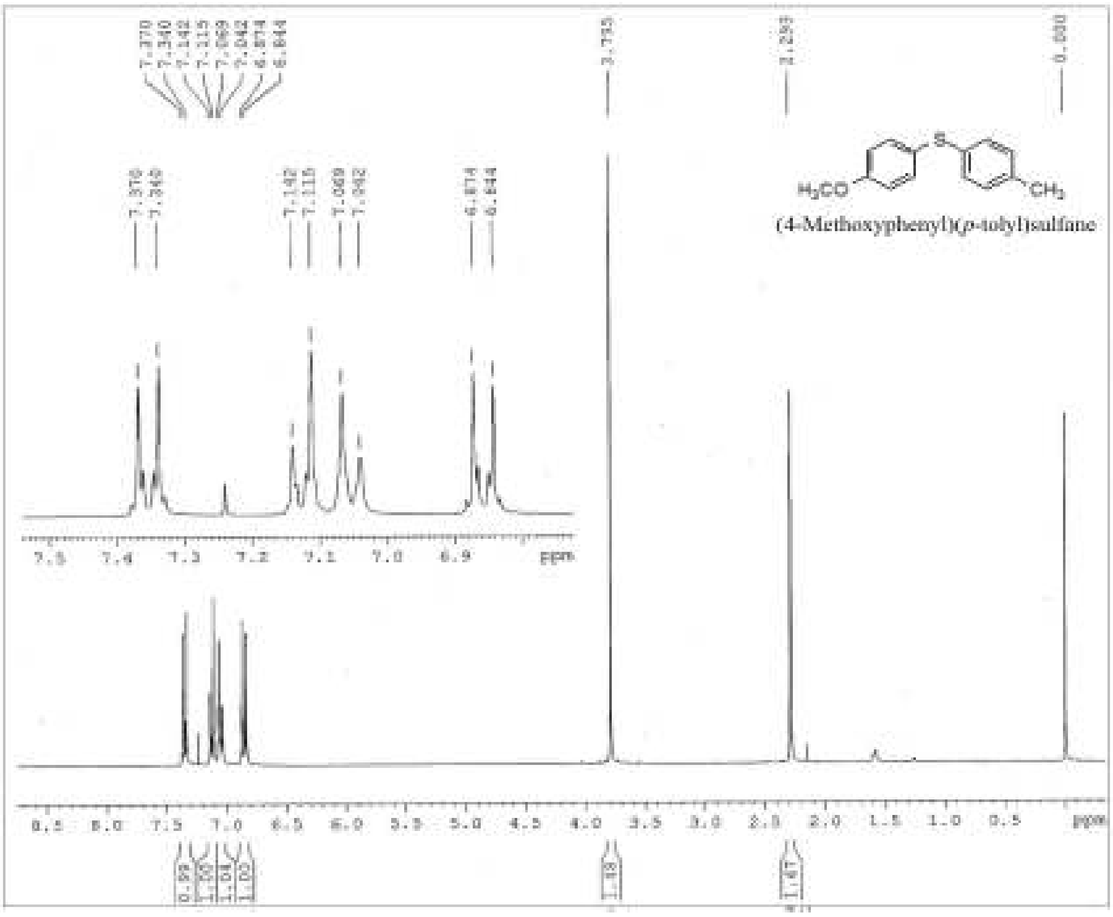

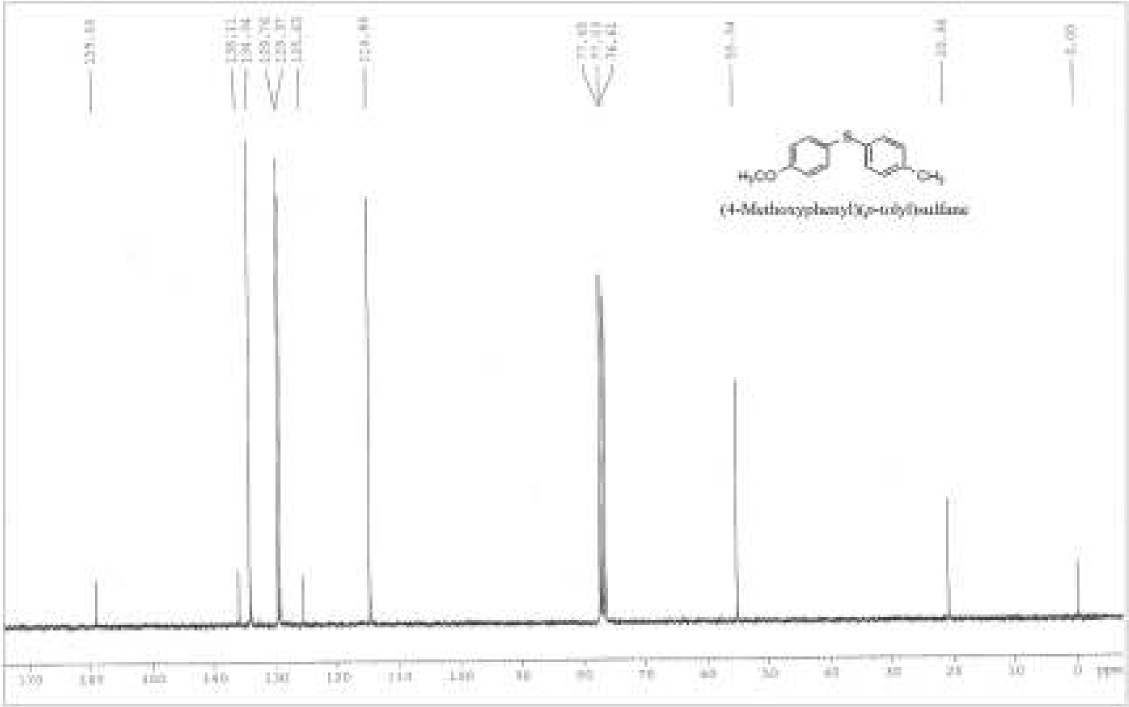

Table 2, entry 9

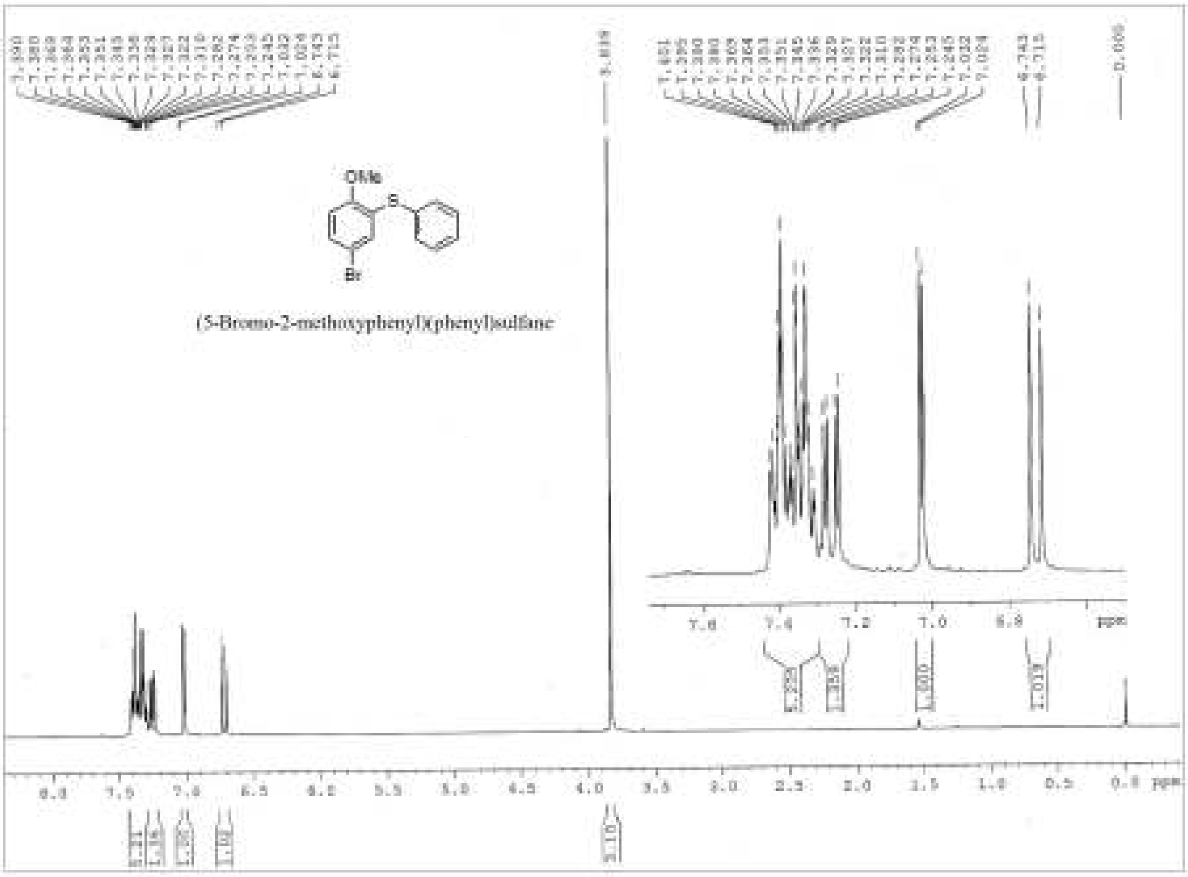

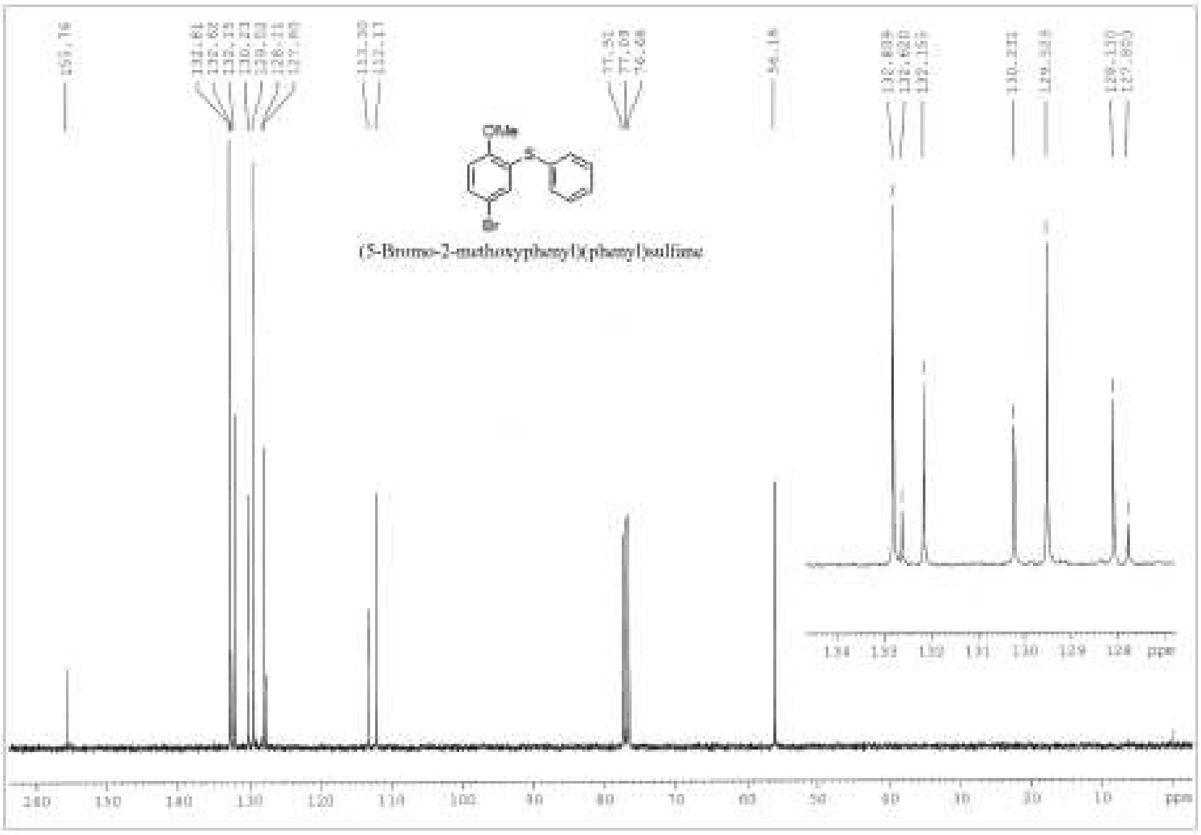

Table 2, entry 10

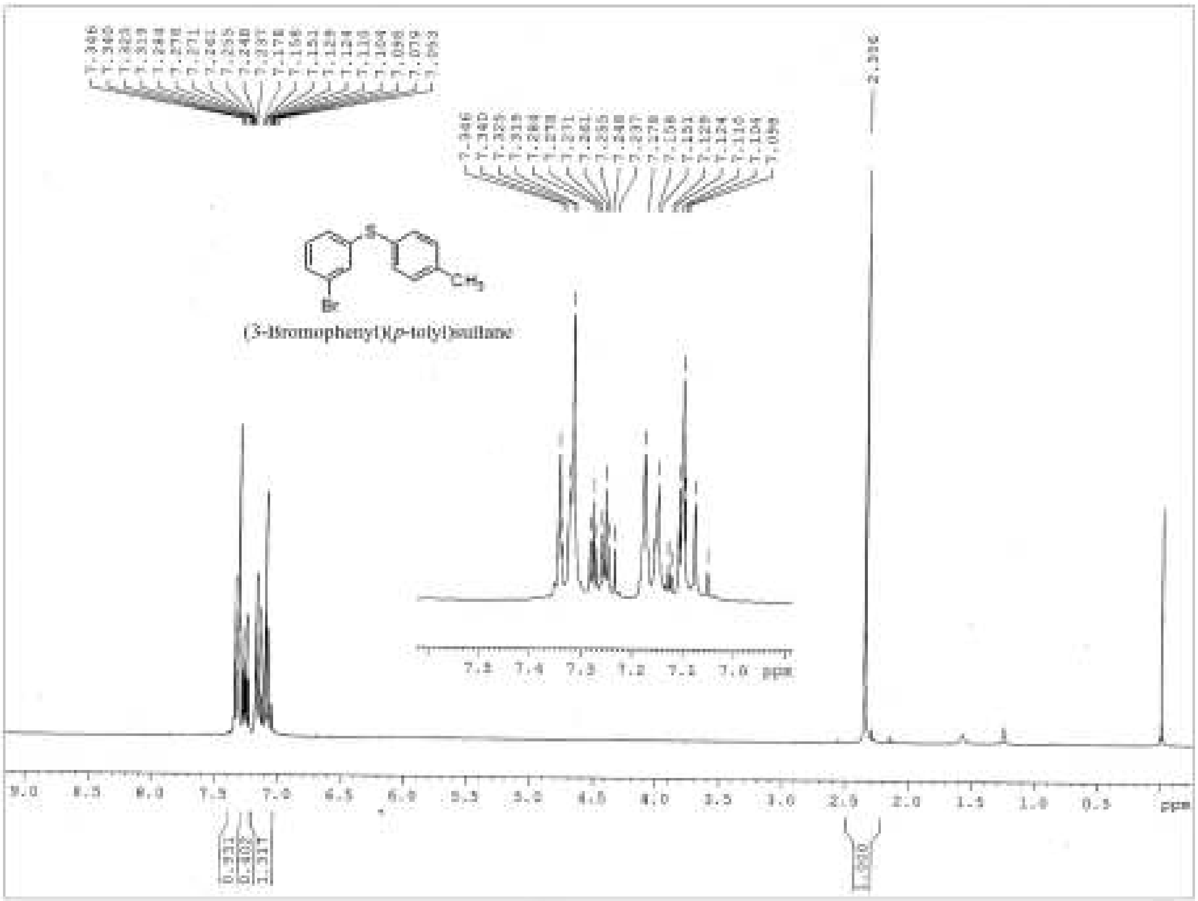

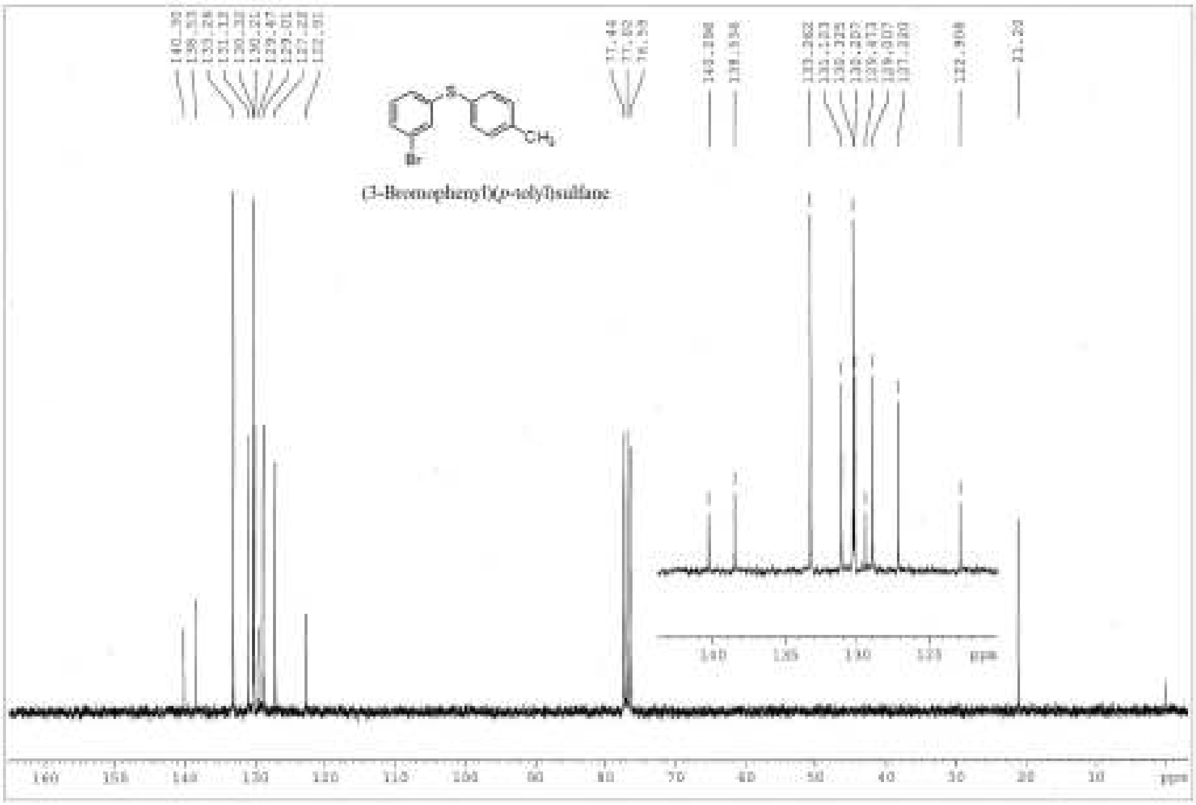

Table 2, entry 11

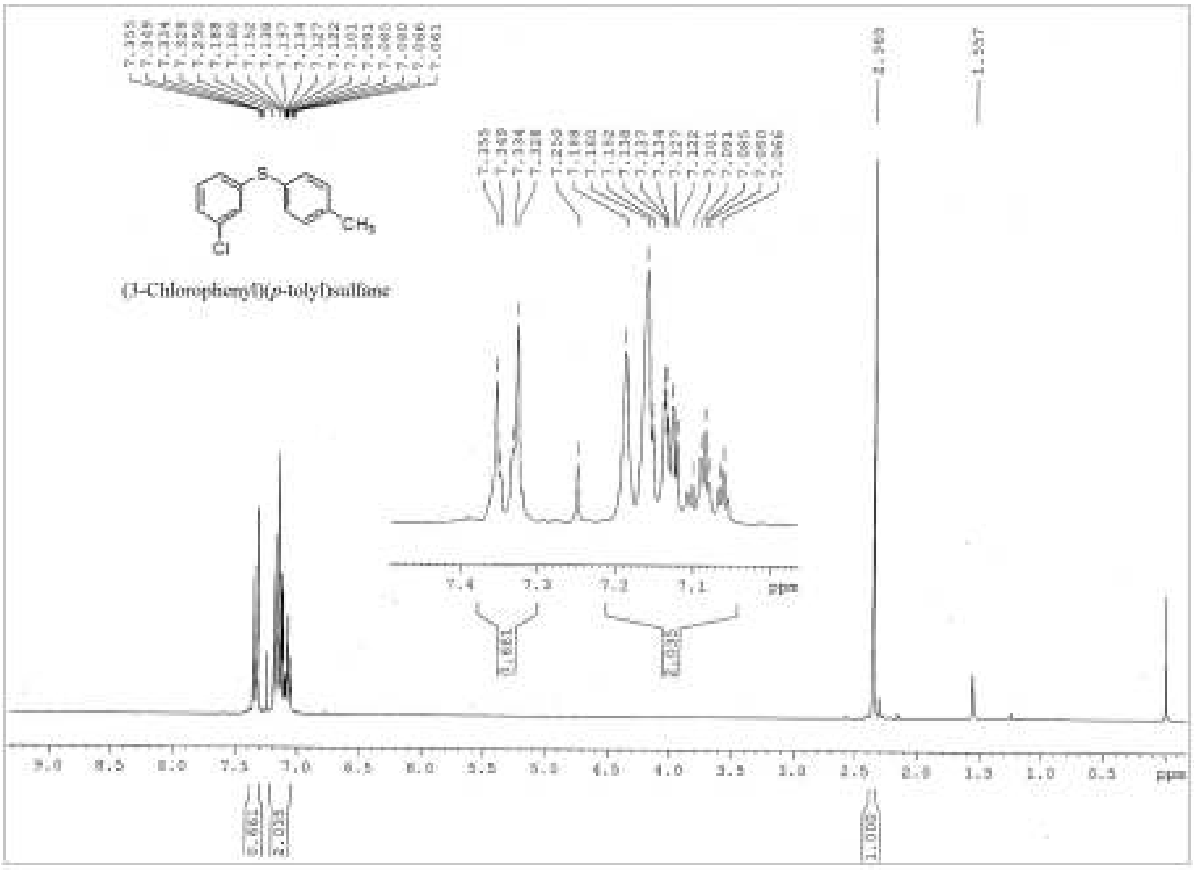

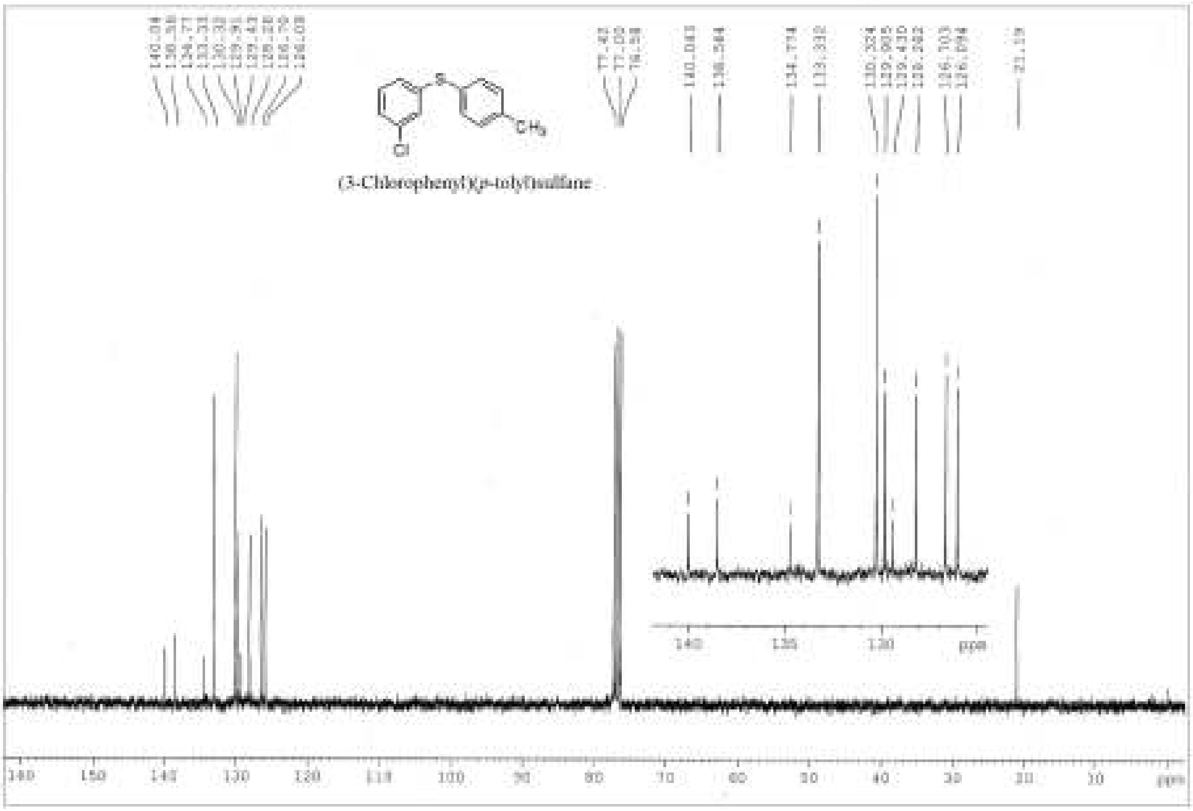

Table 2, entry 12

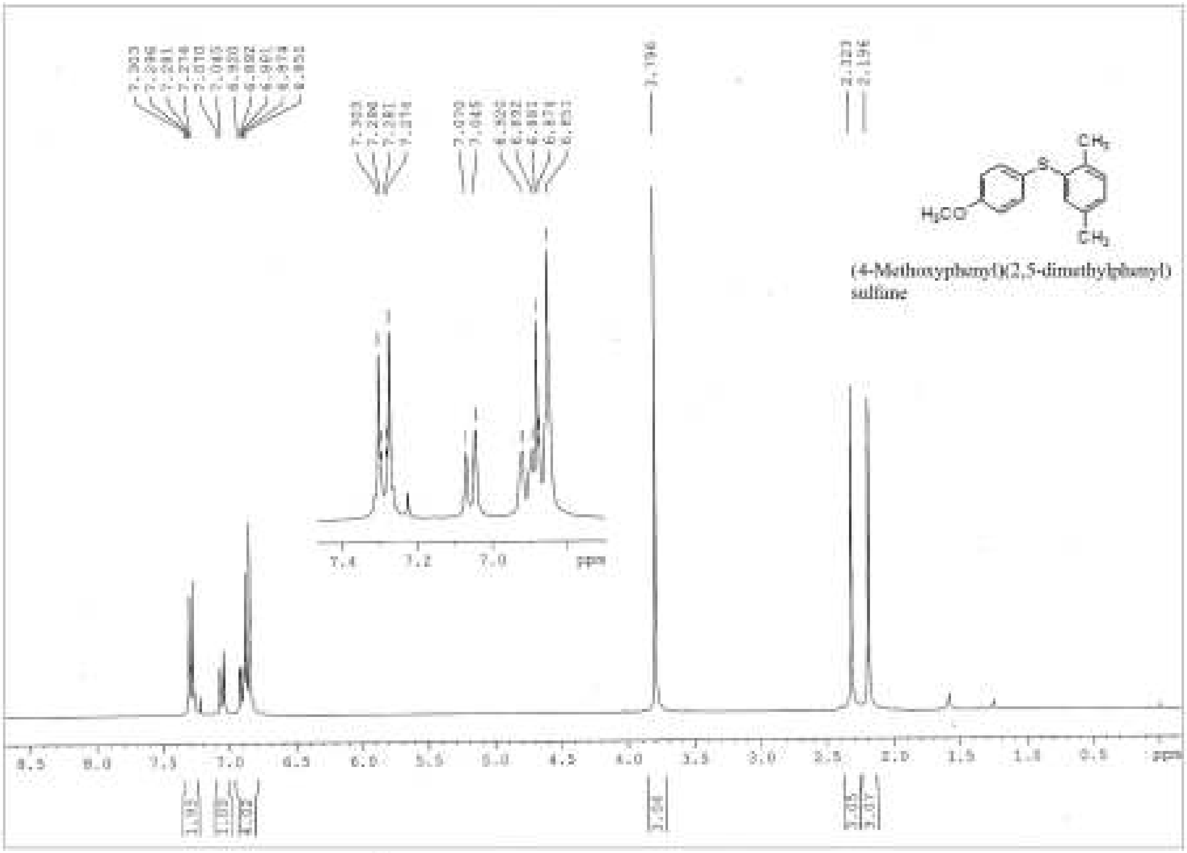

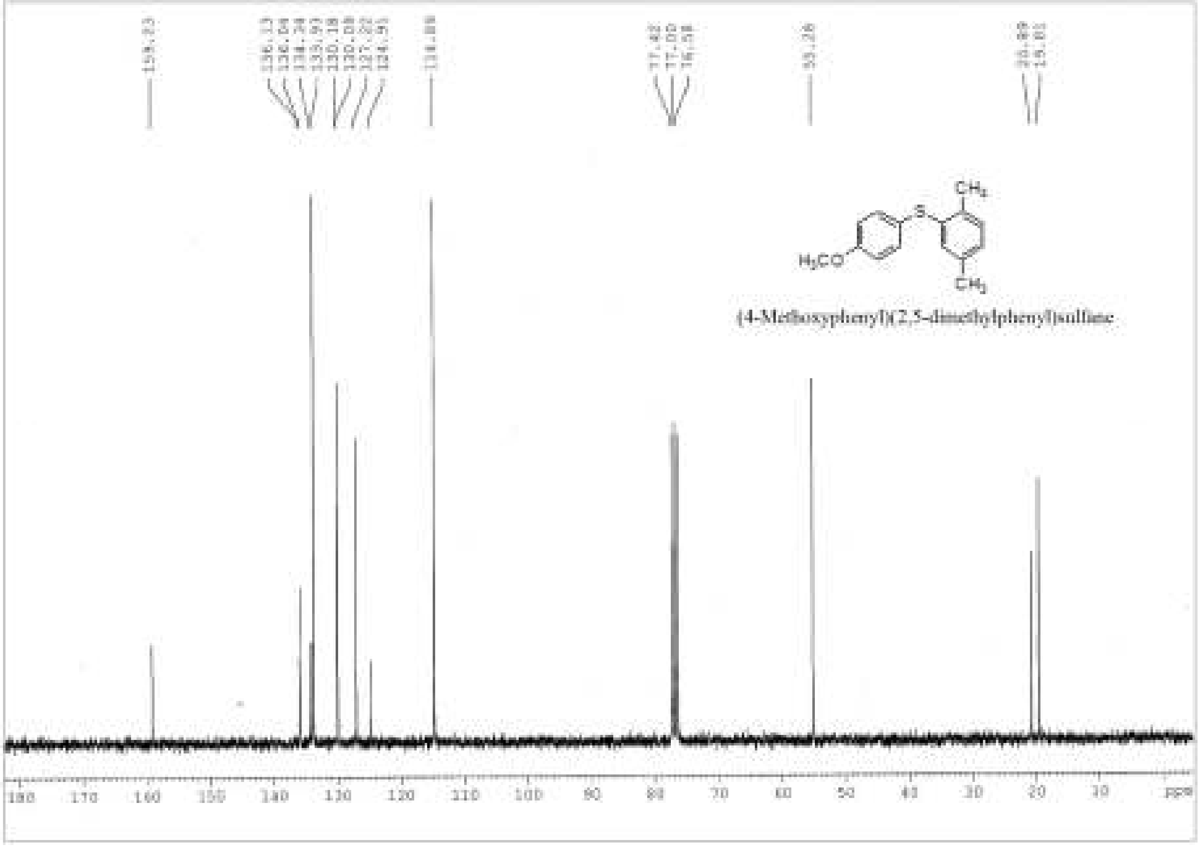

Table 2, entry 13

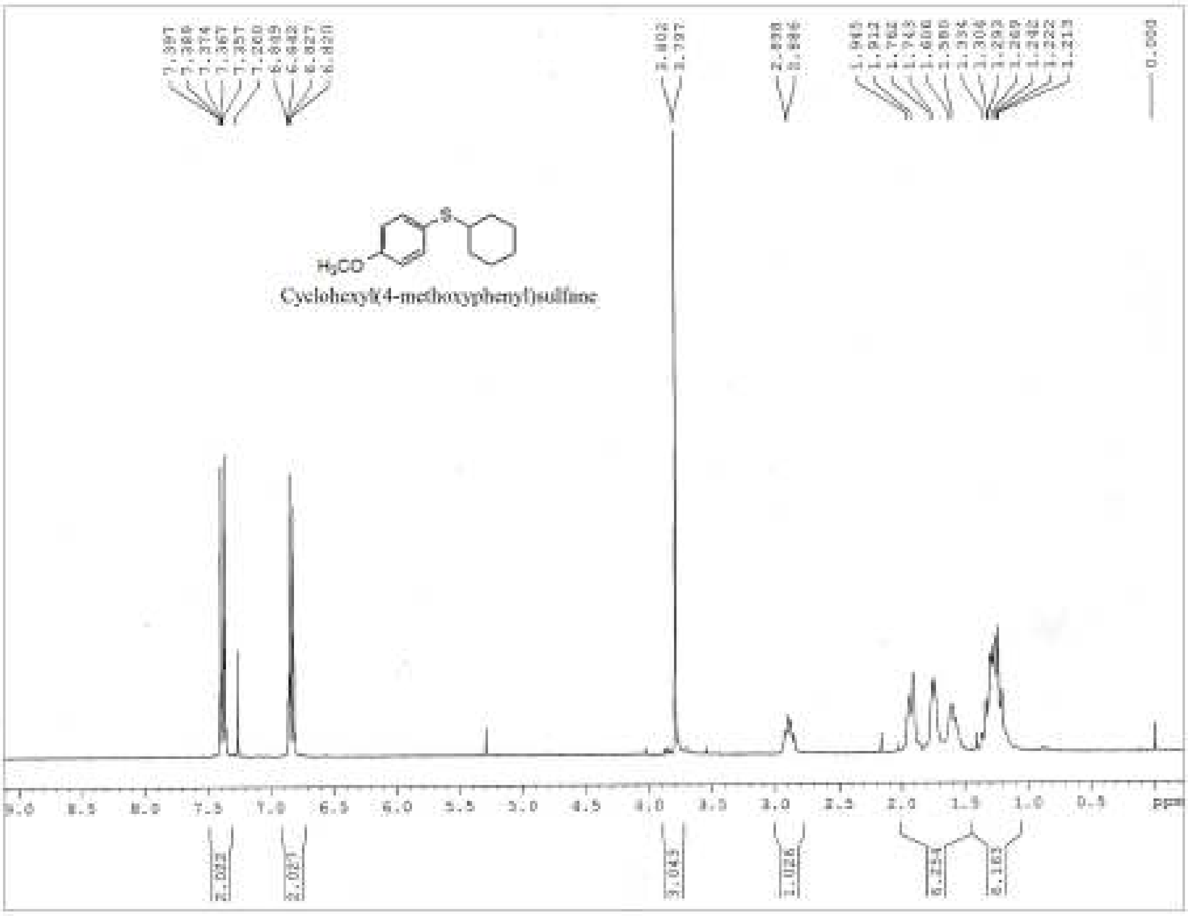

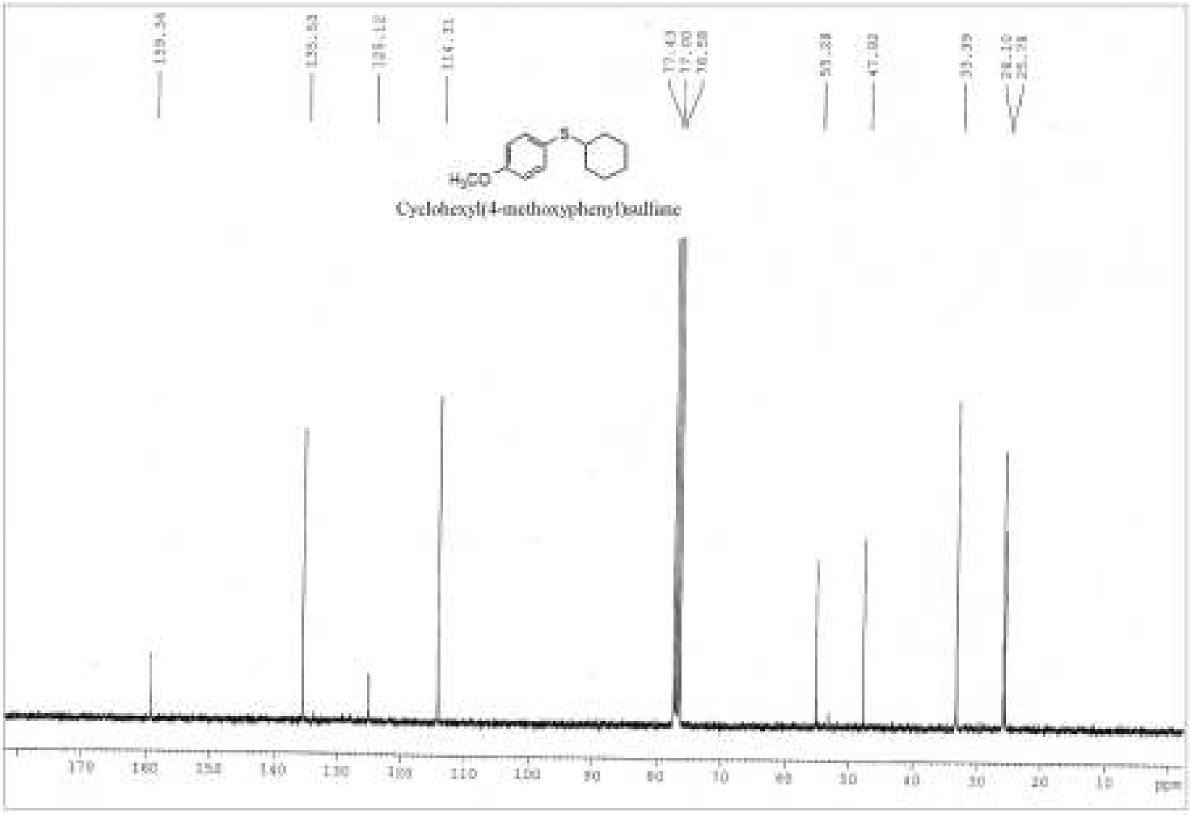

Table 2, entry 14

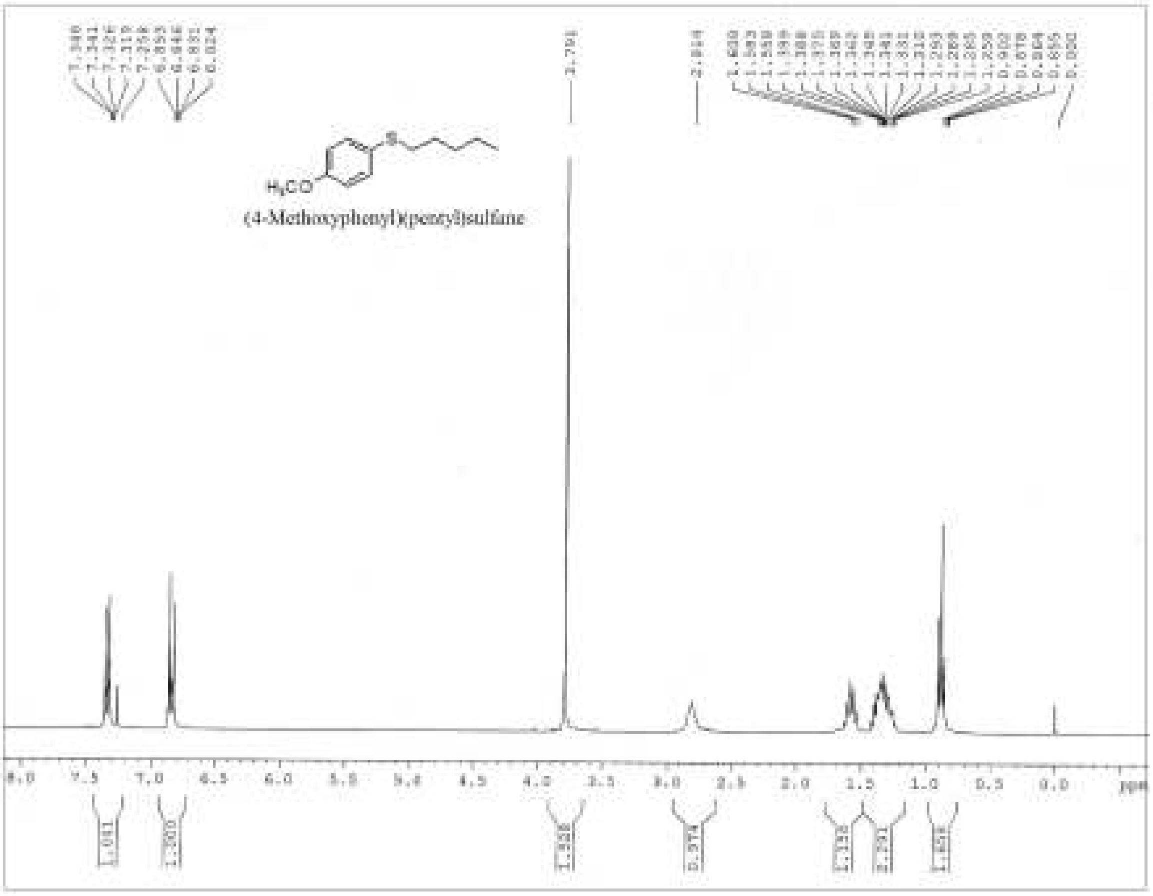

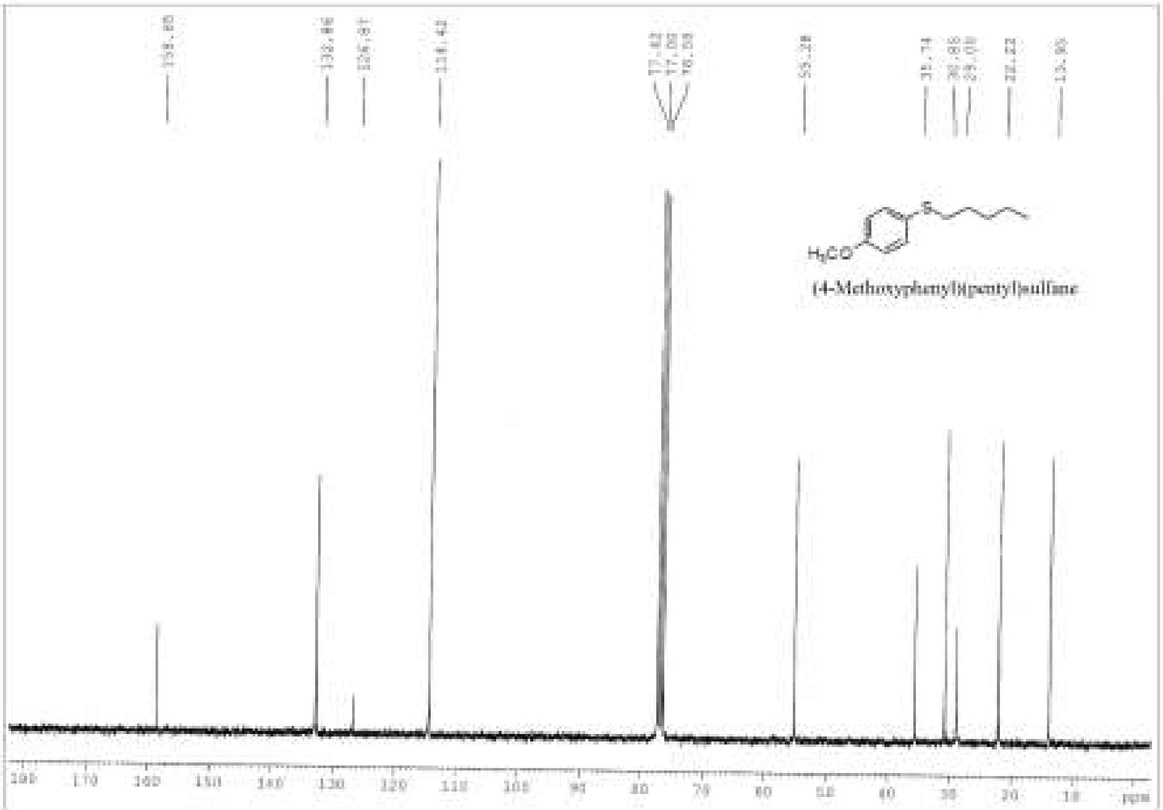

Table 2, entry 15

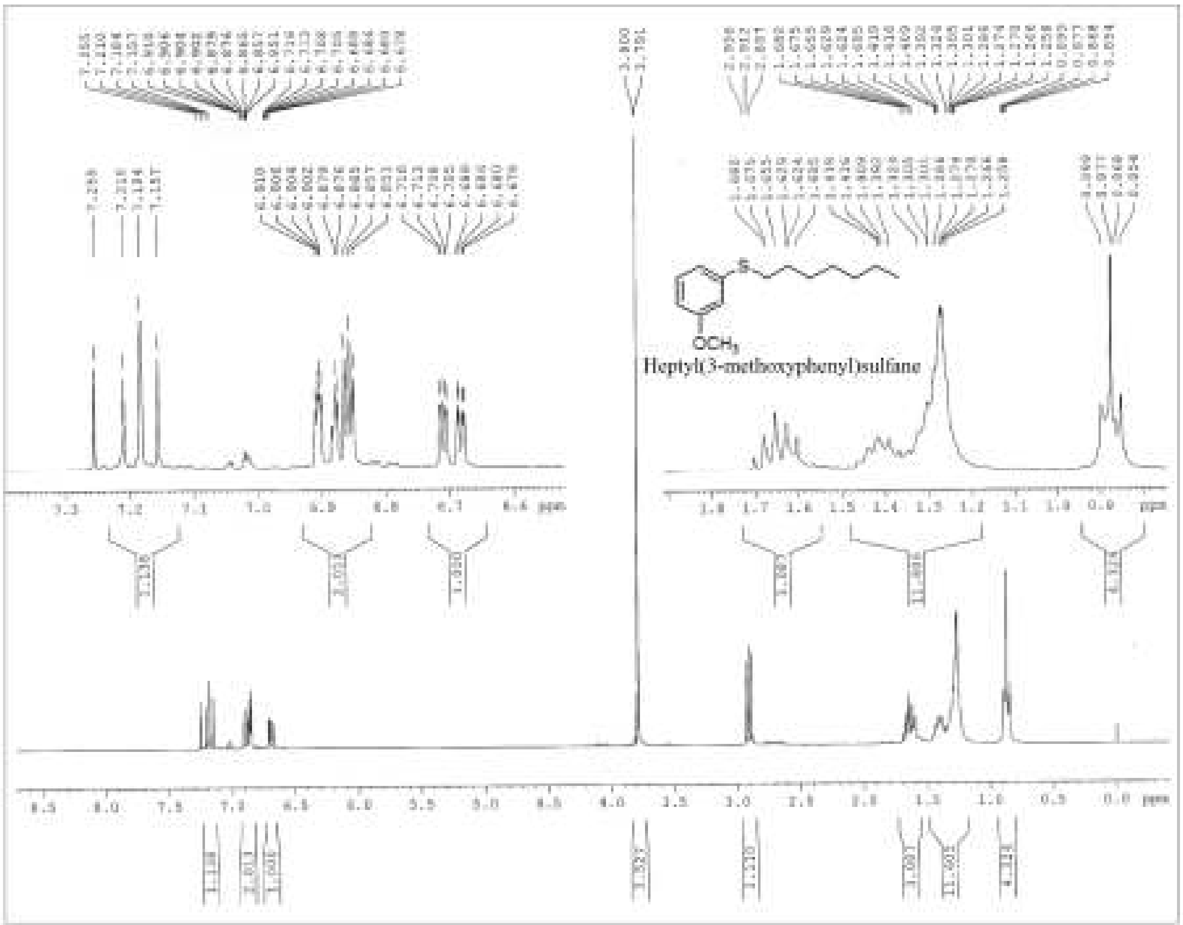

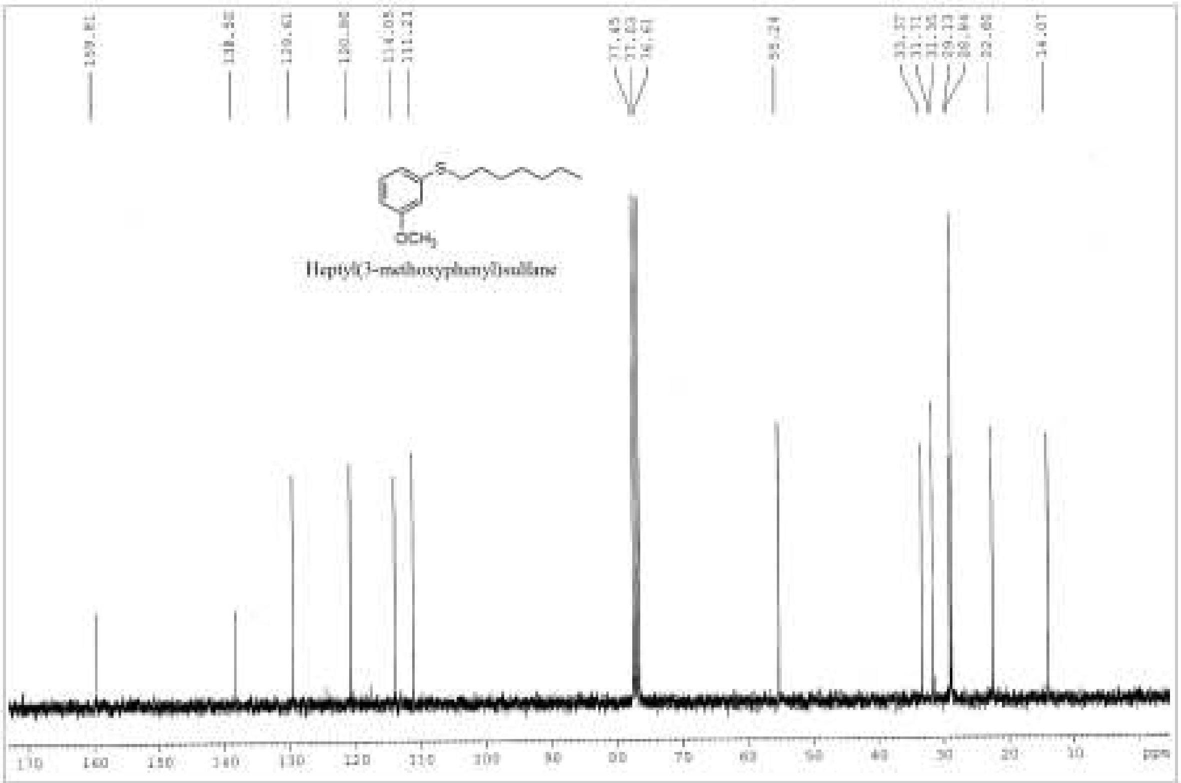

Table 2, entry 16

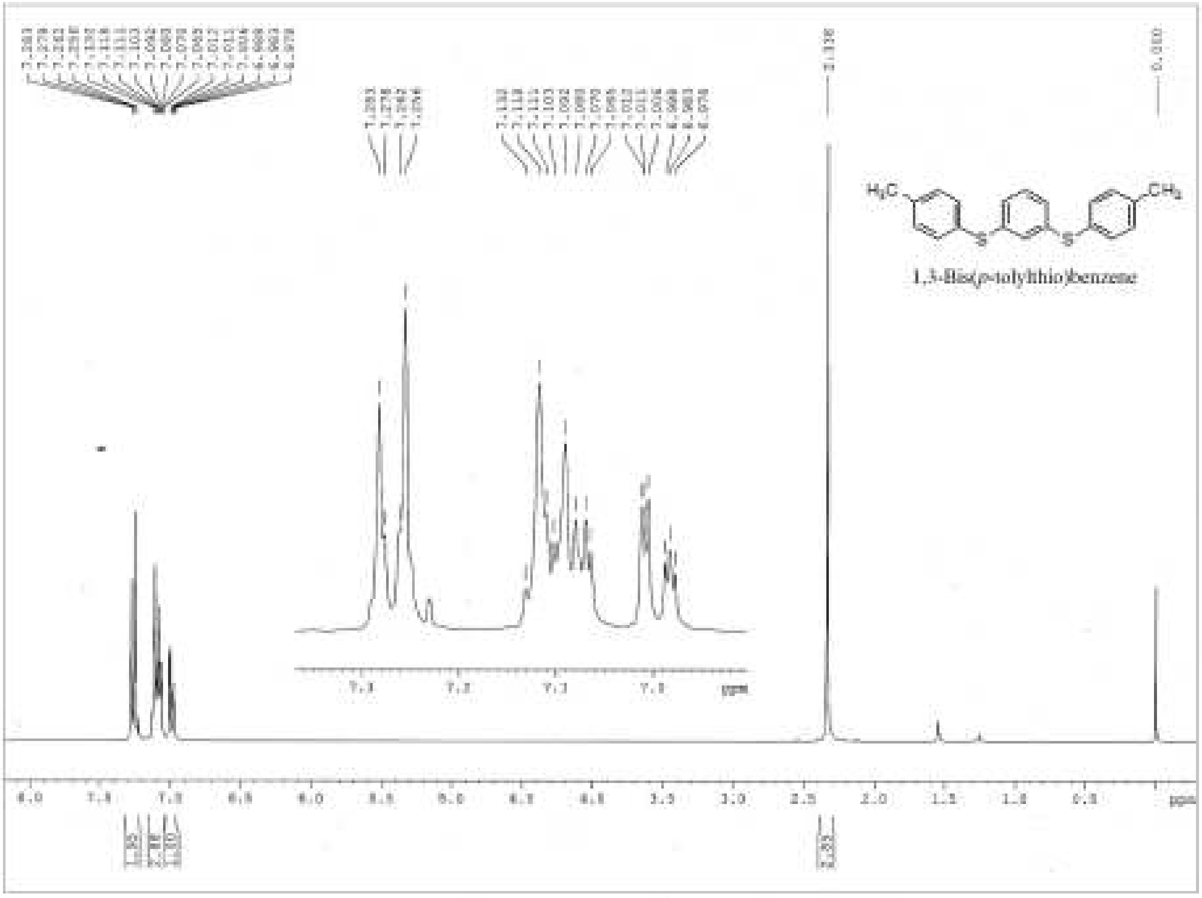

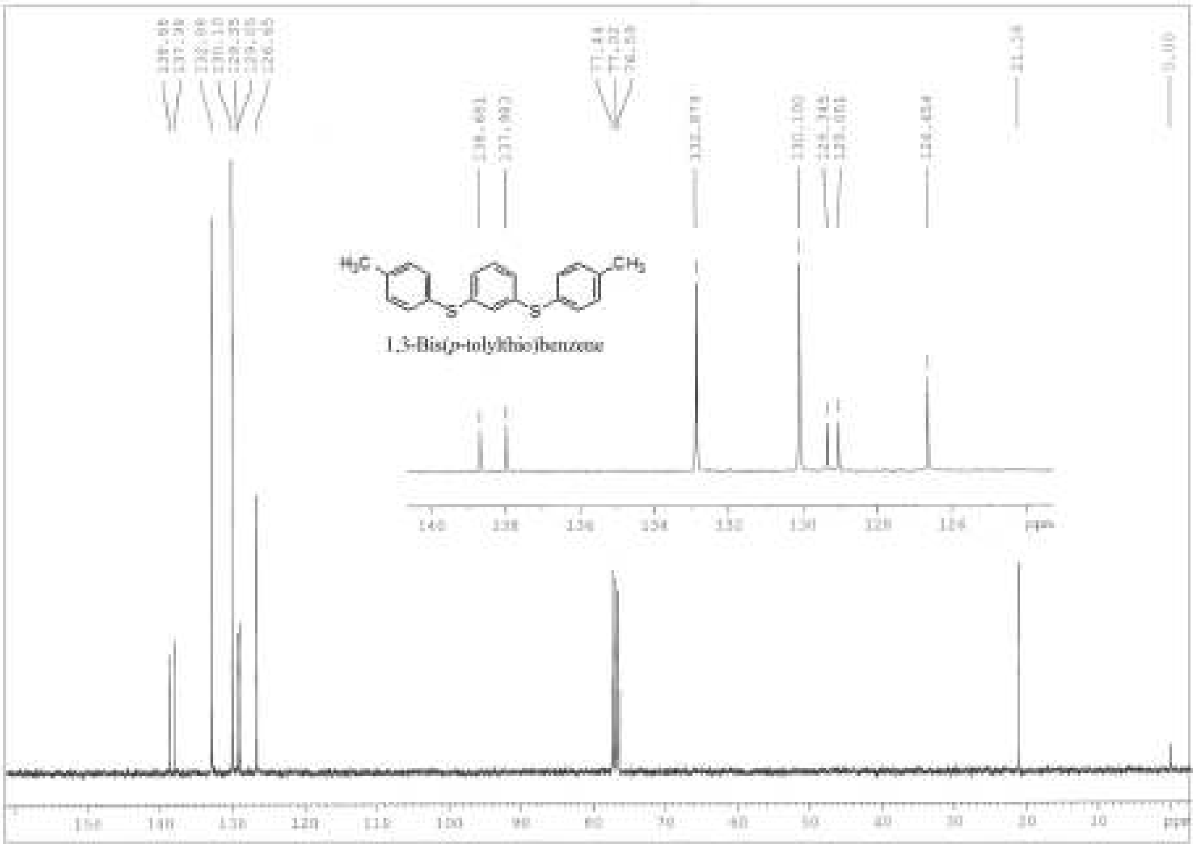

Table 2, entry 17

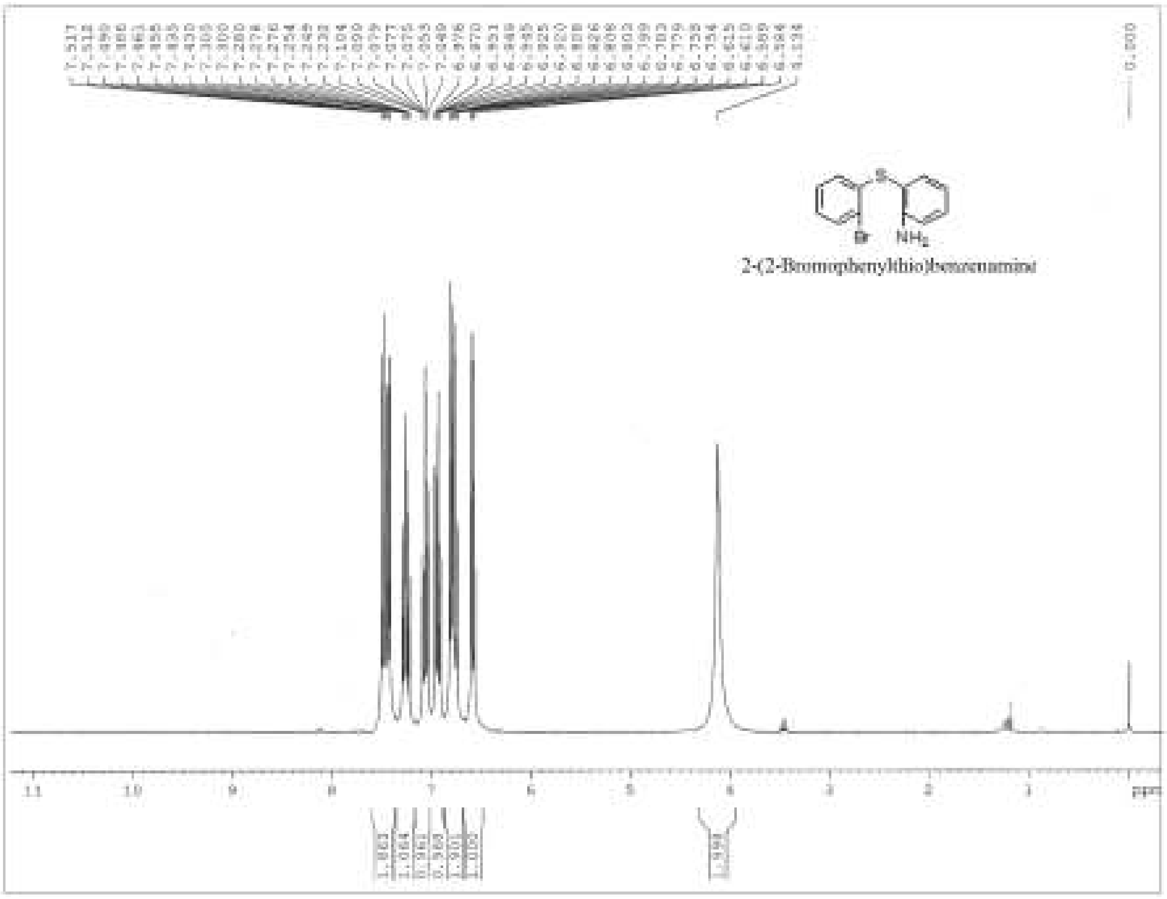

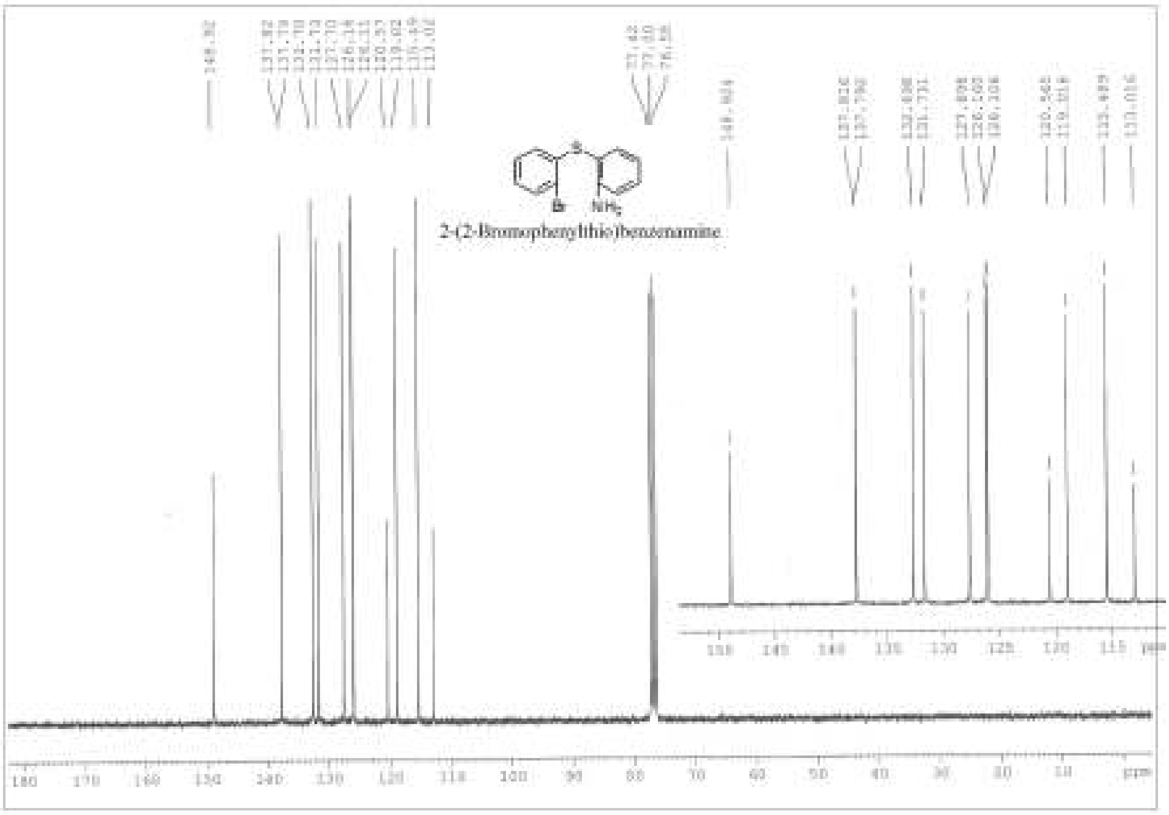

Scheme 1, step 2: Phenothiazine (in DMSO-d6)

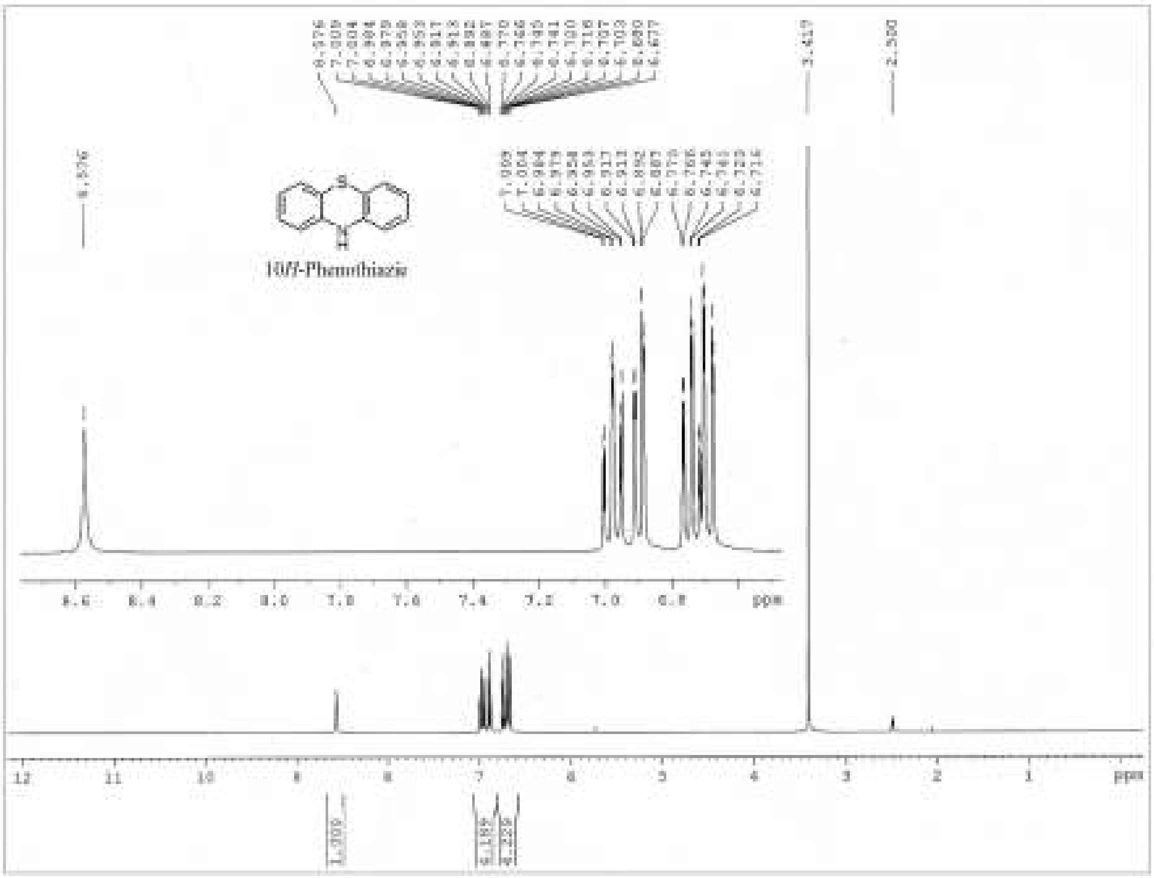

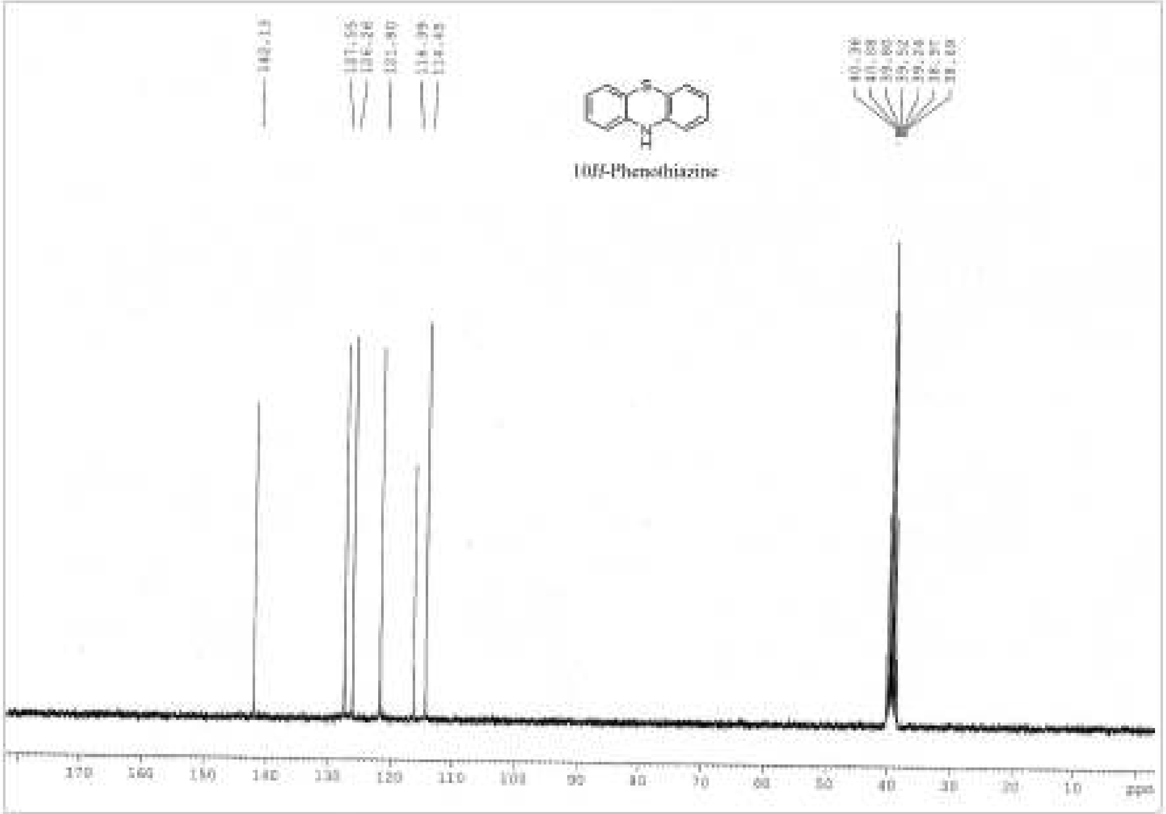

S4. A comparative ^{1}H-NMR spectra for Phenothiazine run in DMSO-d6 and DMSO-d6+D_2O

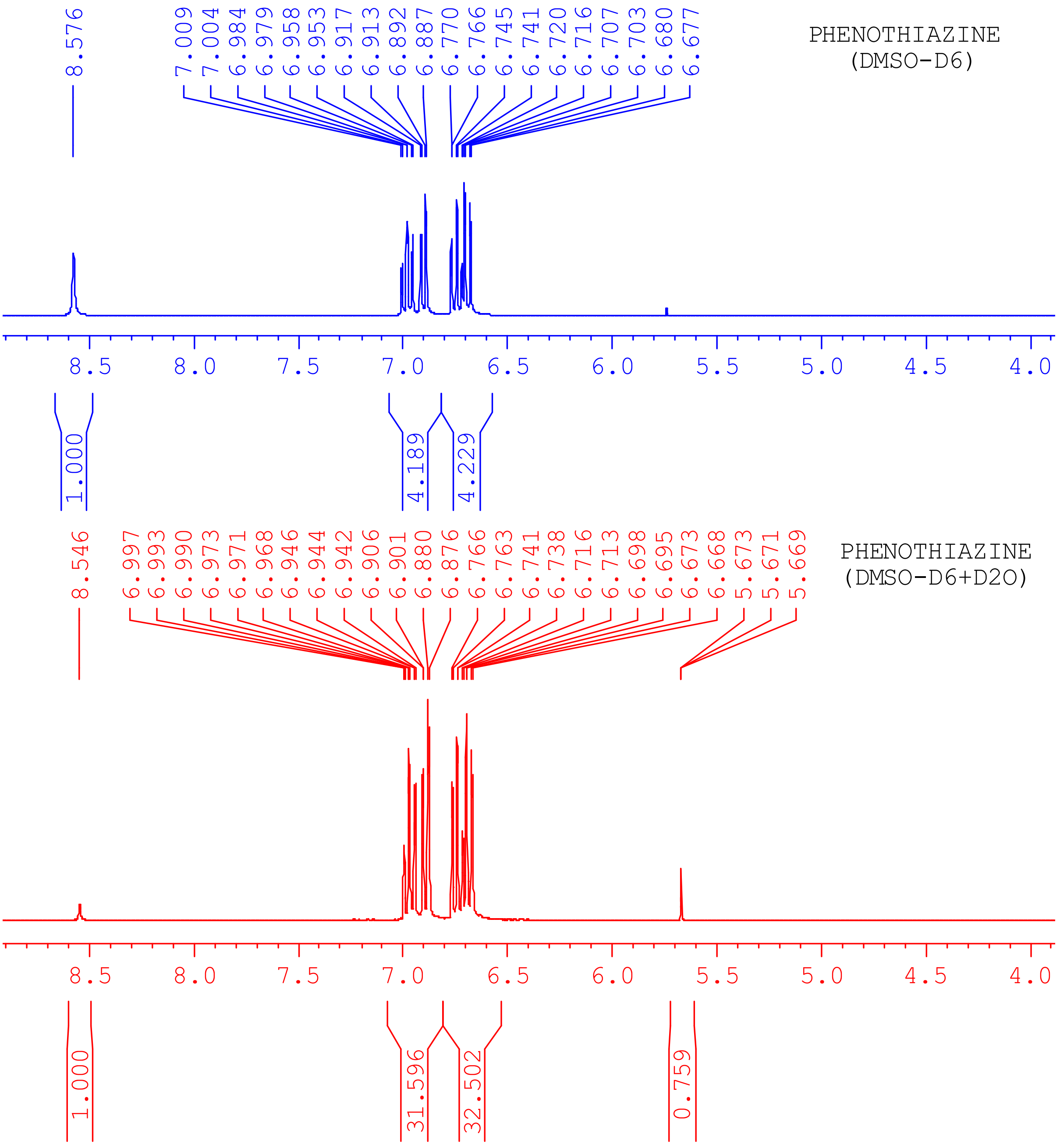

^{13}C–NMR spectra of Phenothiazine in DMSO–d6 (blue) and DMSO–D6+D_2O (red)
