# Supplementary figures and images for: An efficient heterogeneous catalyst (CuO@ARF) for on-water C-S coupling reaction: an application to the synthesis of phenothiazine structural scaffold
Source: Org Med Chem Lett. 2014 Dec 29;4:17. doi: 10.1186/s13588-014-0017-7 (PMC4970439; doi:10.1186/s13588-014-0017-7)

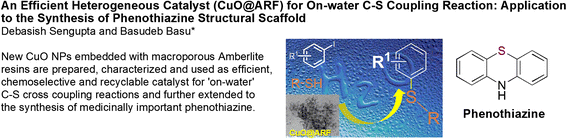

Supplement: Supplementary file 2 — Authors’ original file for figure 1 [file 13588_2014_17_MOESM2_ESM.gif]

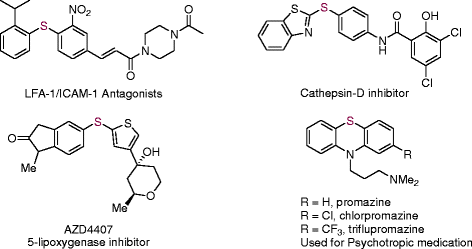

Supplement: Supplementary file 3 — Authors’ original file for figure 2 [file 13588_2014_17_MOESM3_ESM.gif]

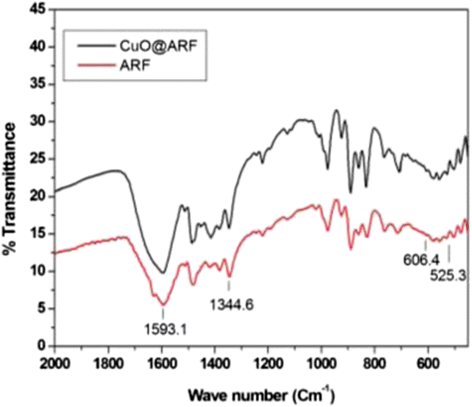

Supplement: Supplementary file 4 — Authors’ original file for figure 3 [file 13588_2014_17_MOESM4_ESM.gif]

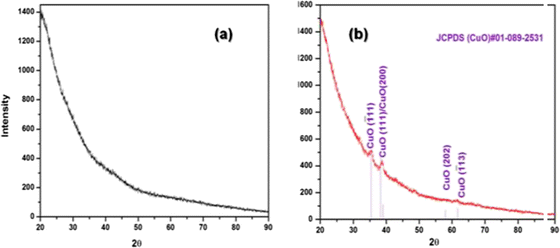

Supplement: Supplementary file 5 — Authors’ original file for figure 4 [file 13588_2014_17_MOESM5_ESM.gif]

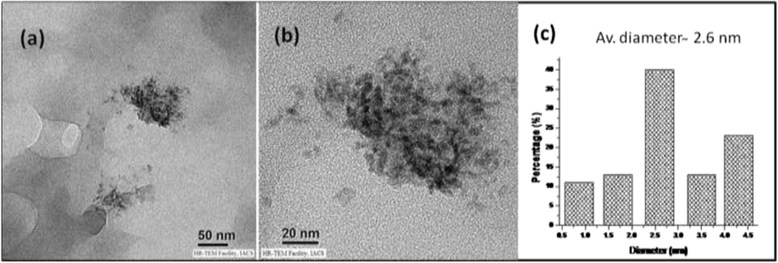

Supplement: Supplementary file 6 — Authors’ original file for figure 5 [file 13588_2014_17_MOESM6_ESM.gif]

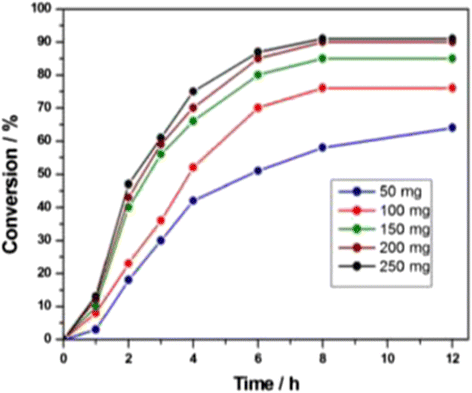

Supplement: Supplementary file 7 — Authors’ original file for figure 6 [file 13588_2014_17_MOESM7_ESM.gif]

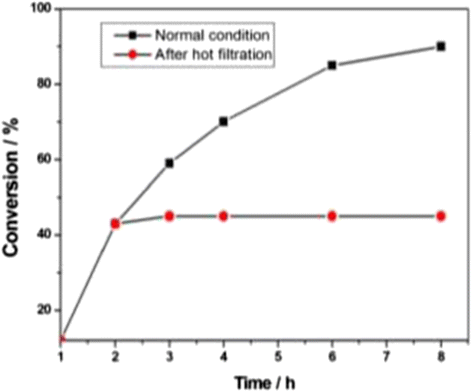

Supplement: Supplementary file 8 — Authors’ original file for figure 7 [file 13588_2014_17_MOESM8_ESM.gif]

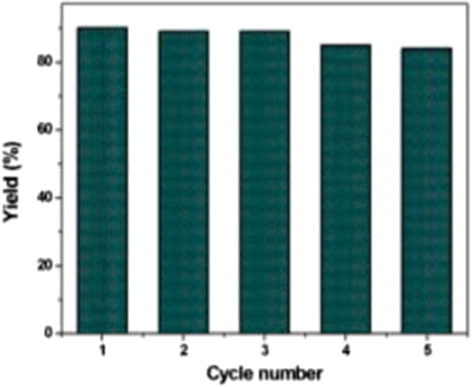

Supplement: Supplementary file 9 — Authors’ original file for figure 8 [file 13588_2014_17_MOESM9_ESM.gif]
